# Advanced Dual‐Ion Batteries with High‐Capacity Negative Electrodes Incorporating Black Phosphorus

**DOI:** 10.1002/advs.202201116

**Published:** 2022-04-27

**Authors:** Jens Matthies Wrogemann, Lukas Haneke, Thrinathreddy Ramireddy, Joop Enno Frerichs, Irin Sultana, Ying Ian Chen, Frank Brink, Michael Ryan Hansen, Martin Winter, Alexey M. Glushenkov, Tobias Placke

**Affiliations:** ^1^ MEET Battery Research Center University of Münster Corrensstraße 46 Münster 48149 Germany; ^2^ Research School of Chemistry The Australian National University Canberra ACT 2601 Australia; ^3^ Institute of Physical Chemistry University of Münster Corrensstraße 28/30 Münster 48149 Germany; ^4^ Institute for Frontier Materials Deakin University Waurn Ponds VIC 3216 Australia; ^5^ School of Materials and Energy Guangdong University of Technology Guangzhou Guangdong 51006 P. R. China; ^6^ Centre for Advanced Microscopy The Australian National University Canberra ACT 2601 Australia; ^7^ Helmholtz Institute Münster IEK‐12 Forschungszentrum Jülich GmbH Corrensstraße 46 Münster 48149 Germany

**Keywords:** alloying, anion intercalation, black phosphorus, dual‐ion batteries, graphite

## Abstract

Dual‐graphite batteries (DGBs), being an all‐graphite‐electrode variation of dual‐ion batteries (DIBs), have attracted great attention in recent years as a possible low‐cost technology for stationary energy storage due to the utilization of inexpensive graphite as a positive electrode (cathode) material. However, DGBs suffer from a low specific energy limited by the capacity of both electrode materials. In this work, a composite of black phosphorus with carbon (BP‐C) is introduced as negative electrode (anode) material for DIB full‐cells for the first time. The electrochemical behavior of the graphite || BP‐C DIB cells is then discussed in the context of DGBs and DIBs using alloying anodes. Mechanistic studies confirm the staging behavior for anion storage in the graphite positive electrode and the formation of lithiated phosphorus alloys in the negative electrode. BP‐C containing full‐cells demonstrate promising electrochemical performance with specific energies of up to 319 Wh kg^–1^ (related to masses of both electrode active materials) or 155 Wh kg^–1^ (related to masses of electrode active materials and active salt), and high Coulombic efficiency. This work provides highly relevant insights for the development of advanced high‐energy and safe DIBs incorporating BP‐C and other high‐capacity alloying materials in their anodes.

## Graphical abstract

Dual‐ion batteries (DIBs) have attracted huge attention as a possible alternative for stationary energy storage. In this study, a black phosphorus–carbon composite is evaluated as a negative electrode material for lithium‐based DIBs for the first time. Full‐cells are designed with practical electrode balancing ratios and compared to graphite || graphite full‐cells.

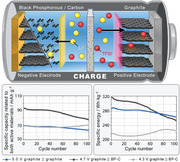

## 1 Introduction

Renewable electricity generation (solar, wind, geothermal, biomass, and waste) has witnessed robust growth in the last decade and its use is expected to keep growing strongly in the next decade. Since some renewable energy sources are intermittent in nature, energy storage solutions are mandatory for enabling their constant usage. Rechargeable batteries have attracted huge attention in the last years^[^ ^1^ ^]^ and the estimated global battery demand in the range of ≈1000 GWh per year and >2600 GWh per year will be required by 2025 and 2030, respectively.^[^ ^2^ ^]^ Concomitantly, changing global policies concerning climate change have also caused a surge in the production of battery‐operated electric vehicles (EVs); 250 million EVs are expected to be on the roads by the end of 2030.^[^ ^3^ ^]^ Lithium‐ion batteries (LIBs) are the most popular batteries, especially for EVs, due to their high energy and power densities, and long cycle life.^[^ ^4^ ^]^ The enormous predicted demand for battery storage combined with limited availability and uneven distribution of lithium and transition metal resources in the world has stimulated researchers to work on alternative battery technologies.^[^ ^5^ ^]^ Rocking‐chair type batteries based on the familiar operating principle of LIBs but using alternative, more abundant cations such as sodium (Na^+^),^[^ ^6^ ^]^ potassium (K^+^),^[^ ^7^ ^]^ calcium (Ca^2+^),^[^ ^8^ ^]^ and magnesium (Mg^2+^)^[^ ^9^ ^]^ have gained increased attention in the recent years. In addition, new types of batteries based on different charge storage mechanisms such as dual‐ion batteries (DIBs)^[^ ^10^ ^]^ have emerged in the last decade. Among various available alternatives, DIBs attract attention due to their forecasted low cost of electrode materials, better environmental compatibility, and the lack of need for transition metals in the positive electrode.^[^ ^10a^ ^]^

The principle of the electrochemical energy storage in DIBs is different from that in LIBs where only Li^+^ ions are involved in the storage process. In the case of DIBs, anions and Li^+^ ions in the electrolyte intercalate simultaneously into the positive (cathode) and negative (anode) electrodes, respectively, during charge and deintercalate from the electrodes into the electrolyte during the discharge process.^[^ ^10^ ^]^ Therefore, the electrolyte anions and cations in DIBs are considered as active species, whereas the Li^+^ ions in LIBs only act as charge carrier. An obvious major difference with LIBs is the presence of an anion hosting cathode.^[^ ^11^ ^]^ In recent years, different classes of anion host materials including organic materials,^[^ ^12^ ^]^ redox‐active polymers,^[^ ^13^ ^]^ metal organic frameworks (MOFs)^[^ ^14^ ^]^ as well as graphitic carbons^[^ ^15^ ^]^ have been studied. Among these, graphite is the earliest, the most studied and most promising material for anion storage by forming acceptor‐type graphite intercalation compounds (GICs). Initially, the reversible electrochemical intercalation of HSO_4_^–^ anions into graphite had been demonstrated for the first time in 1938.^[^ ^16^ ^]^ At present, common anions of organic battery electrolytes such as PF_6 _^–^,^[^ ^17^ ^]^ BF_4_^–^,^[^ ^18^ ^]^ FSI^–^,^[^ ^19^ ^]^ TFSI^–^,^[^ ^20^ ^]^ FTFSI^–^,^[^ ^21^ ^]^ BETI^–^,^[^ ^21a^ ^]^ ClO_4_^–^,^[^ ^22^ ^]^ DFOB^–^,^[^ ^23^ ^]^ and AlCl_4_^– [^ ^24^ ^]^ have been shown to (de‐)intercalate electrochemically into/from graphite. The intercalation of anions occurs at a high operating potential (≥4.5 vs Li|Li^+^) with a reversible capacity up to 150 mAh g^–1^,^[^ ^24^ ^]^ depending on the type of anion,^[^ ^21a^ ^]^ solvent,^[^ ^25^ ^]^ graphite,^[^ ^26^ ^]^ operating temperature^[^ ^27^ ^]^ and applied potential/cell voltage.^[^ ^10a^ ^]^ An attractive combination of a high operating potential, achievable high capacity, recyclability, and elemental abundance makes graphite‐based DIBs a promising sustainable alternative for stationary energy storage. However, due to the high operating potentials of graphite cathodes the electrolyte suffers from high oxidative stress, resulting in poor Coulombic efficiency (*C*_Eff_).^[^ ^27a^ ^]^ The optimization of electrolyte compositions including solvents, salts, and additives has led to crucial improvements in recent years.^[^ ^10^, ^28^ ^]^ Currently, the usage of ionic liquids (ILs) and highly concentrated electrolytes (HCEs) such as, for example, 4 m LiPF_6_ or 3.4 m LiTFSI in dimethyl carbonate (DMC) deliver the most promising performances in DIBs with graphite cathodes.^[^ ^20^, ^25^, ^28^ ^]^ In contrary to ILs, which are often expensive, HCEs are more cost‐effective, enhance the specific capacity and suppress aluminum current collector dissolution.^[^ ^25^ ^]^

Graphite, with its known ability for reversible Li^+^ intercalation, has also been used in the majority of studies of DIBs as anode material.^[^ ^4^, ^10^ ^]^ Such a choice of anode is strongly underpinned by its very successful previous utilization as the anode material of LIBs. Due to the widespread use of graphite in DIBs in literature, such types of batteries are also often called dual‐graphite (DGBs) or dual‐carbon batteries, emphasizing the use of graphite or related carbon structures in both electrodes.^[^ ^20^ ^]^ Nevertheless, graphite is not completely free of limitations as a candidate for the anode. One obvious area for improvement for the anode in DIBs is the specific capacity. While the Li^+^ intercalation capacity of graphite (maximum capacity: 372 mAh g^–1^) is adequate,^[^ ^29^ ^]^ other anode materials with much superior capacities can be identified on the basis of extensive information accumulated on anode materials for LIBs.^[^ ^30^ ^]^ Another complication in the use of graphite anodes is the possibility of Li metal plating on the electrode due to its low operating potential very close to that of Li metal plating and stripping process. This introduces a well‐known safety hazard in lithium‐based batteries in the form of Li metal dendrites,^[^ ^31^ ^]^ however, the tendency to form dendrites is more severe in DIBs due to the possible presence of unbalanced parasitic reactions in the cathode. Severe fading phenomena in LiPF_6_‐based DGBs caused by the unbalanced parasitic reactions on the anode and cathode and assessed via an ion couple inventory model have been reported by Heidrich et al.^[^ ^32^ ^]^ A promising approach for the simultaneous improvement of both safety and energy density of DIBs is to replace the graphite anode with other candidate materials having higher capacities and operating at a somewhat higher potential with respect to Li|Li^+^. While a higher working potential prohibits Li metal plating, a higher specific capacity of the anode can lead to a higher specific energy even despite a lower resulting cell voltage.

Anode materials that operate via the formation of intermetallic phases with Li, via so‐called “alloying” reaction mechanism demonstrate much higher capacities than graphite, as depicted in Figure S1 (Supporting Information). These higher capacities are achieved by electrochemical alloying with Li^+^ instead of traditional Li^+^ intercalation into the interlayer spacings of a layered material.^[^ ^33^ ^]^ To minimize the effects of significant volume changes typical for the alloying materials, they are often prepared in the form of composites of nanoparticles with a secondary carbon component (matrix), and it has been shown that satisfactory cycling stabilities combined with high capacities can be achieved. While these materials have been initially researched in the field of LIBs,^[^ ^34^ ^]^ anodes incorporating alloying materials are also of interest in DIBs. Recently, a number of publications have reported the application of Si‐based,^[^ ^35^ ^]^ Ge‐based,^[^ ^36^ ^]^ and Al‐based^[^ ^37^ ^]^ materials as anodes for DIBs. Si is the material with the highest specific capacity among all alloying candidate materials, which makes it the main target for commercialization in LIBs.^[^ ^38^ ^]^ Its potential drawback in DIBs, however, is the low operating potential, which makes Li metal plating on Si‐containing anodes a realistic possibility. As discussed above, the propensity to accidentally plate Li metal on a low potential anode is higher in DIBs compared to LIBs. In this context, it is worthwhile to look at other alloying candidates that demonstrate high capacities but somewhat higher operating potentials than Si. As it is seen in Figure S1 (Supporting Information), phosphorus, with the theoretical capacity of 2596 mAh g^–1^ has the second highest capacity in this group of materials combined with a higher operating potential (≈0.4–1.2 V vs Li|Li^+^).^[^ ^33^, ^39^ ^]^ Early works on sodium‐based dual‐ion batteries of Yu et al. demonstrated the potential of phosphorus‐based anodes.^[^ ^40^ ^]^ Despite being such an attractive candidate, phosphorus‐containing anode materials have not been comprehensively considered for DIBs utilizing Li^+^ ions and anion intercalation in the graphitic cathode yet.

Here, DIBs with anodes incorporating black phosphorus are experimentally evaluated for the first time. The initial electrochemical characterization of a phosphorus–carbon composite (anode material) and graphite (cathode material) is followed by the evaluation of the corresponding DIB full‐cells with an HCE (3.4 m LiTFSI in DMC). Particular attention is given to the selection of practical capacity balancing ratios for anode and cathode as well as appropriate cell voltage windows and monitoring electrode potential of both electrodes. The key performance indicators of the phosphorus‐graphite full‐cells, including their specific capacity, Coulombic efficiency, specific energy, and energy efficiency^[^ ^41^ ^]^ are evaluated and compared to those of DGBs as well as DIBs with alloying anodes (Si and Ge active phases) described previously in literature.^[^ ^35^, ^36^ ^]^ This timely study provides important initial information relevant to the future design of practical DIB full‐cells with the alloying anode chemistry.

## 2 Results and Discussion

### 2.1 Black Phosphorus–Carbon Composite and Its Electrochemical Analysis

**Figure 1:**
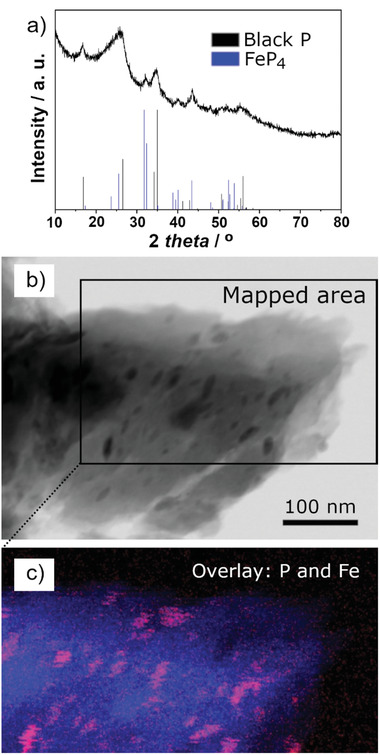
Characterization of BP‐C composite: a) XRD pattern of the material (strong diffraction line positions of black phosphorus and FeP_4_ phases are provided as references); b) bright‐field scanning TEM image and c) overlay of P and Fe STEM‐EDX maps obtained from the highlighted area in (b). Color scheme in the overlay: phosphorus—blue, iron—red.

The XRD pattern of the BP‐C composite prepared using the planetary ball mill is shown in **Figure** **1**a. Graphite used as a carbon precursor in the ball milling procedure is no longer detectable due to its amorphization in the course of ball milling. Furthermore, the strongest (002) reflection of graphite at 26.5° (Inorganic Crystal Structure Database card # 98‐007‐6767) coincides with the XRD reflections from other phases in the pattern. As shown in Figure 1a, the XRD reflections visible in the pattern can be attributed to two phases. The dominant phase is that of crystalline black phosphorus with an orthorhombic structure (ICSD card #98‐002‐3836), and the family of crystalline peaks originating from this phase is observed. The reflections are all significantly broadened, suggesting a very small average crystallite size for black phosphorus. Similar diffraction reflections were observed by Park and Sohn,^[^ ^42^ ^]^ who developed the synthesis method adapted here, and in our previous studies.^[^ ^43^ ^]^ The XRD evidence convincingly demonstrates the formation of nanocrystalline black phosphorus upon milling of red phosphorus under an inert atmosphere. In addition to the XRD signature of black phosphorus, less prominent reflections of another phase, identified as FeP_4_ (ICSD card # 98‐000‐2442), are observed in the pattern. This phase forms in the composite because of a contamination of the material by steel debris originating from the ball milling vial and balls in the process of mechanical milling. The predominantly iron‐based contaminants react with phosphorus in the course of milling to form an iron phosphide, FeP_4_.

As discussed elsewhere in our previous work,^[^ ^43a^ ^]^ the structure of the phosphorus–carbon composite represents small nanoparticles of black phosphorus (consistent with the observed broadening of peaks in the XRD pattern) dispersed homogeneously in the carbon component of the composite. A bright‐filed scanning TEM (STEM) image of a sample and an overlay of P and Fe elemental maps are shown in Figure 1b,c. Comparing the map of phosphorus (blue color) in Figure 1c with the image in Figure 1b, it is obvious that the phosphorus EDX signal is spread over the whole area highlighted in Figure 1b, demonstrating an excellent mixing between phosphorus and carbon components. A more in‐depth analysis by STEM and electron energy loss spectroscopy on the edges of composite particles in such a sample (see ref. [43]) can visualize nanoparticles of black phosphorus with sizes in the range of 1–5 nm. The presence of black phosphorus (and not the original red phosphorus phase) is further confirmed by infra‐red spectroscopy and nuclear magnetic resonance.^[^ ^43a^ ^]^ In addition to phosphorus, discrete Fe‐containing nanoparticles are also observed in an elemental map (red contrast in Figure 1c), confirming the conclusions derived from XRD data.

To quantify the extent of FeP_4_ contamination, an SEM‐EDX quantitative elemental analysis was conducted. The EDX signal was collected from 19 separate areas in the sample, and the results were averaged to obtain the composition of the sample. 47.8 wt% of carbon and 44.9 wt% of phosphorus were measured, as well as 5.1 wt% of oxygen, which originates from the exposure of the material to air during the processes of pellet making and transfer. The measured amount of iron contamination is only 1.8 wt%, comparable with minor contamination with tungsten (1.1 wt%) from the material of the pressing die. The overall percentage of all detected elements (see Table S1, Supporting Information) was 101.9%, indicating a good match of this standard‐based measurement with the expected total. The relative ratio of C and P elements is close to 1:1 (the weight ratio in the mixture of precursors for milling). The results indicate that the FeP_4_ contamination detected in the composite material is of a minor nature and is unlikely to contribute in a substantial manner to the electrochemical properties of the sample.

**Figure 2:**
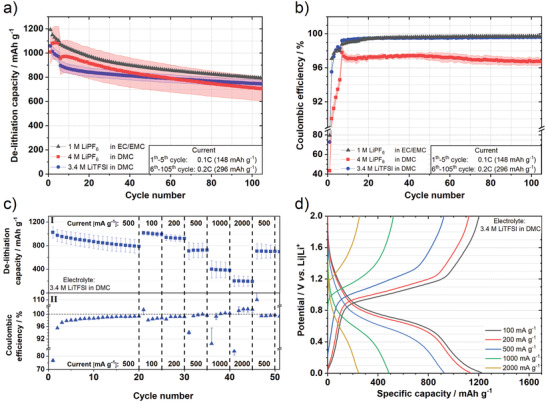
Electrochemical performance of the BP‐C composite in constant current cycling (de‐)lithiation experiments in BP‐C || Li metal cells: a) specific delithiation capacity and b) *C*_Eff_ of long‐term cycling experiments with different electrolytes at 0.2C (1C  =  1484 mA g^–1^) in a voltage range of 0.01–2 V after five formation cycles at 0.1C in two‐electrode coin cells. c) Delithiation capacities (I) and *C*_Eff_ (II) as well as d) corresponding potential profiles at various specific currents with 3.4 m LiTFSI in DMC as an electrolyte operated in three‐electrode cells (WE: BP‐C; CE and RE: Li metal) operated in a potential range of 0.01–2 V versus Li|Li^+^.

The synthesized BP‐C composite material was electrochemically characterized within BP‐C || Li metal cells to evaluate the most promising electrolyte in the intended DIB full cells. Electrodes were prepared as described in the experimental section. Please note, that BP‐containing electrodes were dried at 80 °C to suppress the formation of redox‐active Cu_3_P at the interface between current collector and black phosphorus.^[^ ^44^ ^]^ Because of the known benefits of highly concentrated electrolytes (HCEs) in DIB cells,^[^ ^25^, ^28^ ^]^ the material was cycled in this type of electrolytes (3.4 m LiTFSI in DMC; 4 m LiPF_6_ in DMC) and the performance was compared to that in a state‐of‐the‐art commercial battery electrolyte (1 m LiPF_6_ in EC:EMC, 3:7 by weight; LP57). **Figure** **2**a and Table S2 (Supporting Information) show the specific delithiation capacities of BP‐C in all three electrolytes over 105 charge/discharge cycles. For the LP57 electrolyte, the BP‐C composite material shows a high de‐lithiation capacity of 1194 mAh g^–1^ in the first cycle at 0.1C, which is close to the theoretical capacity of the composite (1484 mAh g^–1^, assuming that the carbon component has a theoretical capacity equal to that of graphite) and is in line with the earlier published results for BP composite materials.^[^ ^43a^ ^]^ After five formation cycles, the capacity drops to 1070 mAh g^–1^ at 0.2C. After 105 cycles the BP‐C material shows a capacity retention regarding the sixth cycle of 74% in the LP57 electrolyte. In comparison, the initial capacity of the BP‐C composite in both HCEs is slightly reduced to 1060 mAh g^–1^ in TFSI‐containing electrolyte and 1009 mAh g^–1^ in 4 m LiPF_6_‐based electrolyte. However, the BP‐C composite shows an increased cycling stability in the 3.4 m LiTFSI (DMC) containing cells with a capacity retention of 83%, whereas with 4 m LiPF_6_ the capacity retention is similar to LP57‐based cells (73%). The capacity retention often directly correlates with the *C*_Eff_ shown in Figure 2b. The BP‐C composite exhibits high initial *C*_Eff_ values of 80 and 73%, in the first cycle in the LP57 and 3.4 m LiTFSI (DMC) electrolytes, respectively, whereas the material suffers from a low initial *C*_Eff_ in the 4 m LiPF_6_ electrolyte (43.5%). After formation, the *C*_Eff_ increases with ongoing cycling for all electrolytes. However, the TFSI‐containing cells show the most stable performance with a high *C*_Eff_ (99.6% in 105th cycle) compared to 4 m LiPF_6_ in DMC (96.7% in 105th cycle).

From literature, it is known that ethylene carbonate (EC)‐free electrolytes will result in a different solid electrolyte interphase (SEI) formation.^[^ ^45^ ^]^ Therefore, it can be assumed that the EC‐free electrolyte based on LiPF_6_ is not able to form an effective SEI at the BP‐C electrode, especially for a high LiPF_6_ salt concentration, which is reflected by the poor *C*_Eff_. Future studies might focus on systematic improvement of the *C*_Eff_ by optimized electrolytes using SEI‐forming additives. With regards to the intended application of this promising anode material in full cells, cycling stability, and *C*_Eff_, especially in the first cycle, are both important factors. Due to its high cycling stability and high *C*_Eff_ 3.4 m LiTFSI in DMC was chosen as the electrolyte for further experiments.

The BP‐C electrodes were cycled via the RE with different specific currents within three‐electrode BP‐C || Li metal cells to evaluate the rate capability in the chosen electrolyte. Considering the design of full‐cells with a suitable capacity balancing, this determination of the practical capacity of the active material at various currents is crucial for a well predictable performance of the full cell. Therefore, the BP‐C composite was first cycled at 500 mA g^–1^ for 20 cycles, corresponding roughly to 0.5C, which matches well with the current applied in the subsequent full‐cell experiments. The specific delithiation capacities and *C*_Eff_ at different specific currents are depicted in Figure 2c. The “artifacts” in *C*_Eff_ for high specific currents (i.e.,>100% at 2000 mA g^–1^) might be a result of the test procedure and cell setup, e.g., because of a not fully delithiated BP‐C electrode. The BP‐C composite shows an initial delithiation capacity of 1021 mAh g^–1^ at 500 mA g^–1^, which decreases to 786 mAh g^–1^ after 20 cycles. The initial *C*_Eff_ is ≈77% concordant with previous results and increases with ongoing cycling to 99.3%. Upon switching to the lowest current (100 mA g^–1^) the specific delithiation capacity increases again to 1017 mAh g^–1^. However, at a highest current of 2000 mA g^–1^, the capacity drops significantly to 200 mAh g^–1^_,_ demonstrating the kinetic limitations of the BP‐C composite material. The initial capacity is fully recovered when the current is brought back to 500 mA g^–1^ afterward.

In general, the *C*_Eff_ increases with higher currents, but drops significantly after applying higher currents and vice versa: A typical potential profile (Figure 2d) of the BP‐C composite material is dominated by a plateau between 0.8 and 0.6 V versus Li|Li^+^ surrounded by sloping regions during lithiation. In the delithiation process, a clear hysteresis can be observed since the most of lithium is released at a plateau between 0.9 and 1.2 V versus Li|Li^+^. Overall, the BP‐C composite shows a promising performance in 3.4 m LiTFSI in DMC as an electrolyte, with a high cycling stability and high *C*_Eff_. This warrants further investigations of this material in the DIB full cells.

### 2.2 Characterization of Graphite as Positive Electrode Material

**Figure 3:**
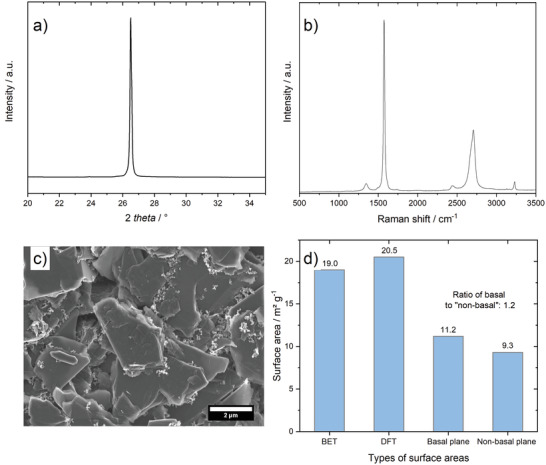
Characterization of the graphite positive electrode material: a) XRD pattern, b) Raman spectrum, c) SEM picture and d) BET surface areas of KS6L graphite. Raman data taken from previous publication.^[^ ^46^ ^]^ SEM picture in c) shows KS6L graphite in a pristine electrode.

For the cathode in DIB full‐cells, a commercial KS6L graphite is used and the characterization data are shown in **Figure** **3**. KS6L graphite shows a strong characteristic (002) reflection at 26.5° in the XRD pattern, which is typical for graphitic structures (Figure 3a). In the Raman spectrum of pristine KS6L (Figure 3b, Raman data taken from previous publication^[^ ^46^ ^]^), strong bands at ≈1580 cm^–1^ and 2690 cm^–1^ can be observed, which can be assigned to G‐band and G´‐band, respectively, which are strong indicators for graphitic structures in carbonaceous materials and are related to the in‐plane stretching vibration of the carbon–carbon double bond.^[^ ^47^ ^]^ Furthermore, a small D‐band at 1350 cm^–1^ is visible, which relates to carbon atoms at the graphene edges or different kinds of disorders.^[^ ^47^, ^48^ ^]^ The intensity ratio of D‐band to G‐band (*I*_D_/*I*_G_) is ≈ 0.06, demonstrating the highly graphitized structure and is also typical for graphites.^[^ ^47a^ ^]^ KS6L graphite has a flake‐like morphology with a D90 particle size of 8.5µm (Figure 3c). As already shown in our previous publication, the used KS6L graphite possesses a high surface area (19 m^2^ g^–1^) with a high amount of non‐basal planes (9.3 m^2^ g^–1^; Figure 3d), indicating promising properties for anion intercalation. This abundance of non‐basal planes can be determined via gas adsorption measurements calculating the DFT adsorption potential. The correlation of nonbasal planes and electrochemical performance was discussed in previous publications.^[^ ^15^, ^49^ ^]^

**Figure 4:**
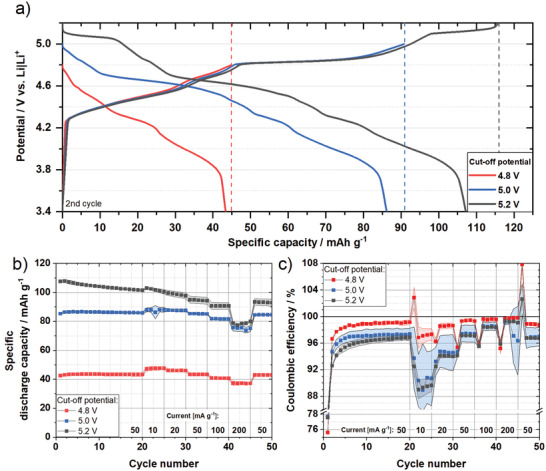
Electrochemical performance of graphite as a positive electrode material in constant current cycling charge/discharge experiments in graphite || Li metal cells (three‐electrode configuration; half‐cell setup).: a) Potential profiles of graphite at 50 mA g^–1^ of the second cycle, b) specific discharge capacity and c) *C*_Eff_ of graphite WE at various specific currents and different upper‐cut‐off potentials (potential window: 3.4‐X versus Li|Li^+^) with 3.4 m LiTFSI in DMC as electrolyte.

The KS6L graphite material was also electrochemically characterized to evaluate the practical capacity for electrode balancing for DIB full‐cells as well as to determine the optimum upper cut‐off potential. Therefore, graphite || Li metal cells with 3.4 m LiTFSI in DMC were assembled and electrochemically characterized with different upper cutoff potentials (4.8 V; 5.0 V; 5.2 V vs Li|Li^+^). The potential profiles of these TFSI‐based GICs are shown in **Figure** **4**a. It can be clearly seen that the profiles are dominated by a series of plateaus. Indeed, acceptor‐type GICs are famous for their staging behavior and their characteristic plateaus at various potentials.^[^ ^25^, ^27^, ^50^ ^]^ Based on earlier studies, it is well known that the formation of stage‐IV and stage‐III GICs occurs first in the beginning of the intercalation process. At ≈4.8 V versus Li|Li^+^ a stage II‐TFSI‐GIC evolves, followed by a plateau, which is caused by a stage II–stage I transition, ending up at 5.0 V versus Li|Li^+^. At ≈5.2 V versus Li|Li^+^, the stage‐I‐TFSI GIC is fully developed, corresponding to a theoretical stoichiometry of (TFSI)C_20_.^[^ ^15^, ^27^ ^]^ Due to this staging behavior the upper cut‐off potential has a strong influence on the electrochemical performance. Figure 4b,c displays the specific discharge capacity b) and the *C*_Eff_ c) at different specific currents. With respect to the BP‐C characterization, the graphite WE were first cycled at 50 mA g^–1^ for 20 cycles, corresponding roughly to 0.5 C, and were afterward (dis)charged at different specific currents to evaluate the rate capability. At 4.8 V versus Li|Li^+^ a capacity of 43 mAh g^–1^ is achieved, while the capacity doubles (85 mAh g^–1^) when a cutoff potential of 5.0 V versus Li|Li^+^ is used due to the formation of a stage‐I GIC. By increasing the upper cutoff potential up to 5.2 V versus Li|Li^+^, an even higher discharge capacity of 108 mAh g^–1^ is reached due to a nearly fully developed stage‐I TFSI‐based GIC. In terms of different specific currents, the graphite positive electrodes show a high rate capability. At the highest current of 200 mA g^‐1^ (≈2C) 78% (4.8 V), 86% (5.0 V), and 77% (5.2 V) of the capacity related to the lowest current are still achievable for all three different cut‐off potentials, respectively. In terms of cycling stability, the graphite WE shows a high capacity retention at 4.8 and 5.0 V, whereas a slight capacity fading is visible after 100 cycles for 5.2 V versus Li|Li^+^ (Figure S2, Supporting Information). Due to the high upper charge potentials, irreversible reactions like oxidative electrolyte decomposition can occur, which causes a lower *C*_Eff_ at higher cut‐off potentials. At lower specific currents the *C*_Eff_ drops to ≈90% at high potentials (5.0 V and 5.2 V vs Li|Li^+^), suggesting continuous irreversible side reactions such as the oxidative decomposition or an ongoing internal redox reaction between the solvent and GIC. Nevertheless, a *C*_Eff_ of 97% (5.0 V and 5.2 V) and 99% (4.8 V) is achieved in the 20^th^ cycle at 50 mA g^–1^, indicating the effective passivation of the aluminum current collector by the LiTFSI‐based HCE. The initial *C*_Eff_ is ≈75% at 4.8 V and 77% at 5.0 V as well as 5.2 V versus Li|Li^+^, respectively, which is in a similar range to the initial *C*_Eff_ of the BP‐C composite. Regarding the eventual full‐cell application and the design of high‐energy DIB cells, a high specific capacity of the cathode as well as a high *C*_Eff_ are both important factors to reach high specific energy and stable cycling performance. Therefore, 5.0 V as well as 5.2 V versus Li|Li^+^ were selected as promising cutoff potentials for further design of DIB full‐cells to investigate the impact of both targeted cutoff potentials.

### 2.3 Investigation of BP‐C || Graphite Full‐Cells

In the next step, the results of the graphite WE as well as those of the BP‐C WE from potential‐controlled half‐cell studies were considered to combine both electrode materials in graphite || BP‐C full‐cells and to investigate their electrochemical performance. When thinking about DIB full‐cells, some important aspects have to be taken into account. First, it should be decided which potential windows the negative and the positive electrodes should be exposed to during the full‐cell's cycling. These ranges can be influenced by electrode capacity balancing and the cell voltage window.^[^ ^51^ ^]^ However, both parameters also have an impact on each other. Therefore, a desirable electrode balancing was determined first. In this study, we refer to the capacity‐based balancing as the N/P ratio, to be concise. Considering different N/P ratios for DIB full‐cells, one has to keep in mind that DIB full‐cells often suffer from fading mechanisms which are caused by anion trapping or Li^+^ ion trapping ending up in strong capacity drop or Li metal plating and related safety issues.^[^ ^32^ ^]^ Therefore, an N/P ratio of >1 was used to minimize the risk of Li metal plating. However, with increased N/P ratio the specific capacity as well as the specific energy related to the mass of both active materials decrease, which is discussed in more detail below. As a result, an N/P ratio of 1.2:1 was used, which is a compromise between the still maintained enhanced safety and total cell capacity. For electrode balancing, the practical specific charge capacities in the third cycle from the previous half‐cell studies at 0.5C (for graphite: capacities at 5.2 V vs Li|Li^+^ are used here) were utilized, as the initial interphase formation processes of the materials have been largely completed.

**Figure 5:**
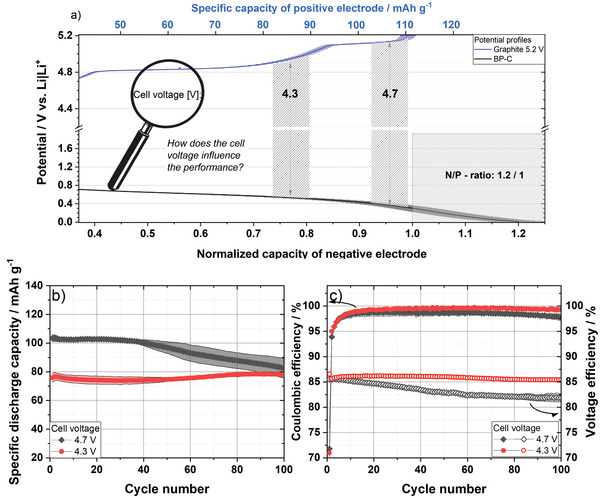
Electrochemical performance of graphite || BP‐C cells in constant current cycling charge/discharge experiments (two‐electrode configuration, full‐cell setup). a) Scheme of matched full potential profiles of graphite and BP‐C from the third charging step (lithiation/anion intercalation) and the influence of different applied cell voltages with an error margin of 5% and a capacity deviation of 10% (transparent area). b) Specific discharge capacity and c) *C*_Eff_ as well as voltage efficiency of graphite || BP‐C full‐cells operated between 2.0 V and different upper cutoff cell voltages (4.3 or 4.7 V) at 50 mA g^–1^ with 3.4 m LiTFSI in DMC as electrolyte. Specific capacities and currents are related to the mass of the positive electrode.

The charge potential profiles of a BP‐C anode as well as those of a graphite cathode are plotted in **Figure** **5**a, normalized to an N/P ratio of 1.2:1. In addition to minimizing Li metal plating, the chosen N/P‐ratio leads to a further beneficial effect. The phosphorus electrode initially cycles in the plateau area of the potential profile (>0.5 V vs Li|Li^+^), whereas the sloping area (<0.5 V vs Li|Li^+^) is avoided. As a result, the reproducibility should be increased for a particular cell voltage due to a lower effect of possible deviations, as can be seen from the error bars of the potential profiles with an assumed capacity‐related deviation of 10% in Figure 5a. Two different upper cut‐off cell voltages (4.3 and 4.7 V) are illustrated with broad vertical lines in the scheme including a 5% error margin to estimate the impact of the targeted electrode potentials and accompanying targeted specific capacities expected in DIB full cells. Based on this plot, it can be understood that the upper cell voltage limits of 4.3 and 4.7 V are representative for graphite cathode cutoff potentials of ≈5.0 and ≈5.2 V versus Li|Li^+^, respectively, and these were chosen as the upper cut‐off potentials of interest based on the previous half‐cell results from graphite || Li metal cells and BP‐C || Li metal cells.

#### 2.3.1 Influence of Cell Voltage on DIB Cell Performance

Figure 5b shows the specific discharge capacities of graphite || BP‐C cells cycled at 50 mA g^‐1^ (≈0.5 C) without any formation cycles in a cell voltage range between 2 V and 4.3 V or 4.7 V, respectively. All values are related to the mass of the positive electrode. As expected, a high upper cutoff voltage of 4.7 V leads to a specific discharge capacity of 103 mAh g^–1^ in the first cycle. After 50 cycles, a capacity of 97 mAh g^–1^ is still reached; however, the capacity subsequently drops to 82 mAh g^–1^ in the 100^th^ cycle. At a cell voltage window of 2 and 4.3 V, an initial discharge capacity of 76 mAh g^–1^ is achieved and a very stable cycle behavior over 100 cycles can be observed. These achieved values go in line with the predicted capacities in Figure 5a, demonstrating the success of the electrode/cell design assumptions. Furthermore, the predicted effect of an increased deviation for 4.7 V (see Figure 5a) can also be seen in the cycling data (Figure 5b). In terms of *C*_Eff_, which is shown in Figure 5c, an initial *C*_Eff_ of 71% and 72% is reached for a cell voltage between 2 and 4.3 V and 2 –4.7 V, respectively. For both voltage windows, the *C*_Eff_ increases in a similar manner to 99.2% (4.3 V) and 98.7% (4.7 V) in the 20^th^ cycle. However, the *C*_Eff_ drops slightly in case of a higher cell voltage of 4.7 V after 80 cycles (100^th^ cycle: 97.7%), which goes in line with previous results from graphite || Li metal cells (Figure S2, Supporting Information). In addition to the *C*_Eff_, the voltage efficiency (*V*_Eff_) is an important indicator analyzing the electrochemical performance. Both graphite || BP‐C full‐cells show an initial *V*_Eff_ of ≈85% which is mainly caused by the hysteresis of the BP‐C negative electrode during discharge (compare Figure 2d). The *V*_Eff_ decreases with ongoing cycling to 82% in the 100^th^ cycle (2.0–4.7 V), whereas the *V*_Eff_ remains nearly constant over 100 cycles for the smaller voltage window (2.0–4.3 V). In summary, the graphite || BP‐C full‐cell shows a high cycling stability in a voltage range of 2–4.3 V, whereas a widened cell voltage window of 2–4.7 V leads to a higher specific capacity but also to a more significant decay in capacity and *V*_Eff_.

**Figure 6:**
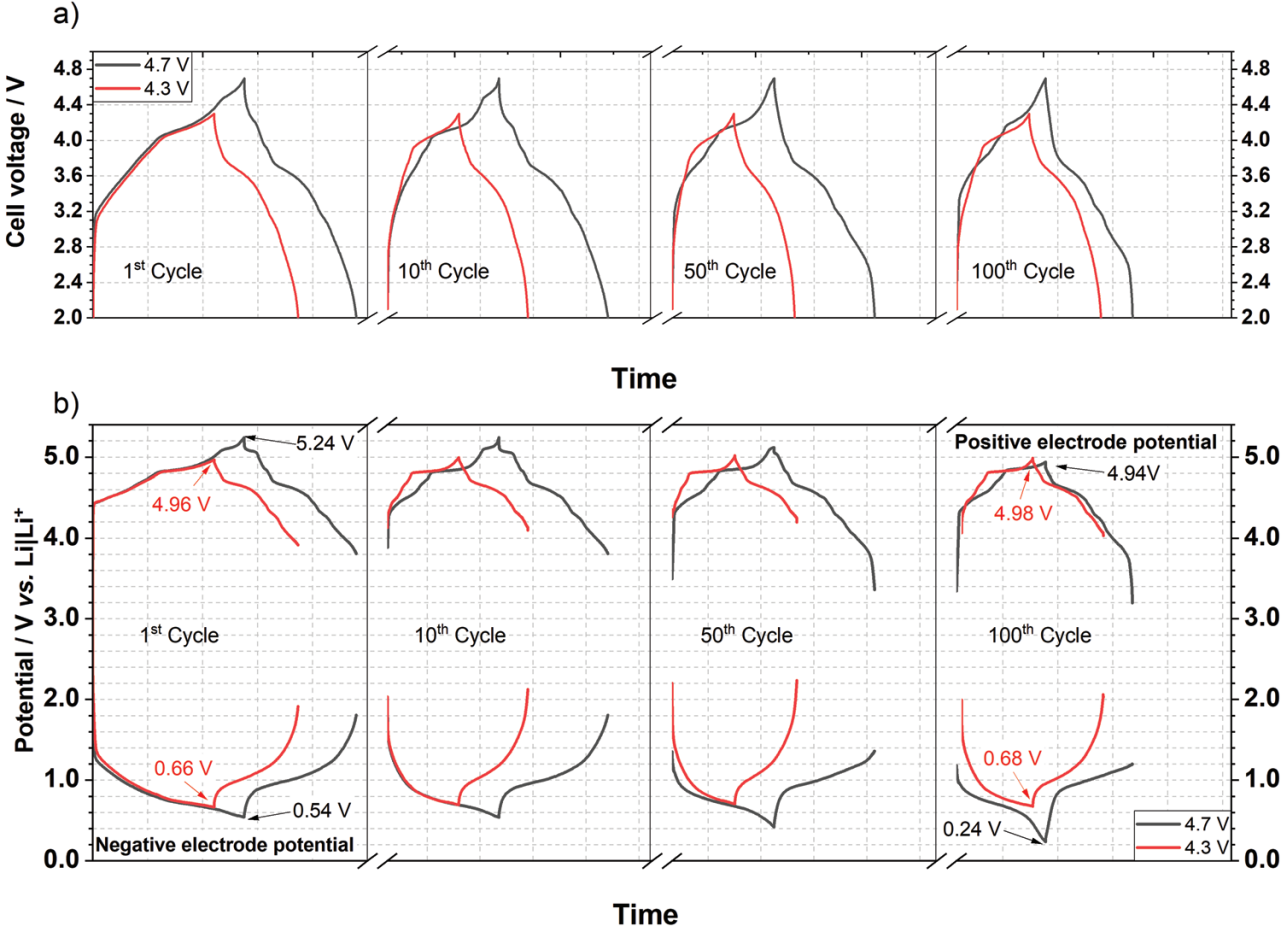
a) Cell voltage and b) electrode potential curves in different cycles of graphite || BP‐C cells (three‐electrode configuration; full‐cell setup) operated between 2 and 4.3 V or 2  and 4.7 V at a current of 50 mA g^–1^ (related to the mass of the positive electrode) with 3.4 m LiTFSI in DMC as electrolyte.

Three electrode cells with both cell voltage windows were cycled to monitor the potentials of both negative and positive electrodes with the assistance of a Li metal RE to gain more insights about the fading mechanism of the full cell. A comparison of the electrochemical performance of two‐electrode and three‐electrode cell setups is shown in Figure S3 (Supporting Information). The courses of cell voltage and electrode potentials of graphite || BP‐C cells for the 1^st^, 10^th^, 50^th^, and 100^th^ cycles are displayed in **Figure** **6** for both cell voltage windows. As it follows from Figure 6, for the cell operating between 2 and 4.3 V, a positive electrode potential of 4.96 V versus Li|Li^+^ is reached during the first charge, whereas the phosphorus‐containing negative electrode's potential decreases to 0.66 V versus Li|Li^+^, which all goes in line with the assumptions made on the basis on the results from Li metal cells in half‐cell setup (Figure 5a).

Both potential curves remain nearly constant for 100 cycles showing no Li metal plating, demonstrating the high cycling stability shown in Figure 5b. For the cell operating at 2–4.7 V, the graphite positive electrode reaches a potential of 5.24 V versus Li|Li^+^ in the first cycle, whereas the phosphorus negative electrode is charged to 0.54 V versus Li|Li^+^. With ongoing cycling, it can be observed that both electrode potentials drop to lower values during charge. However, during discharge, the negative electrode is not fully delithiated (1.2 V vs Li|Li^+^; 100^th^ cycle), whereas the graphite positive electrode is already fully discharged reaching 3.2 V versus Li|Li^+^ (100^th^ cycle). The course of both electrode potentials over 100 cycles provides a hypothetical possibility for Li^+^ ion trapping in the negative electrode during cycling. This phenomenon was described by Heidrich et al. as one possible fading mechanism for DIB full‐cells, which could lead to a Li metal plating in ongoing cycles followed by capacity fading.^[^ ^32^ ^]^ Both observed behaviors can be explained by a comparison of the *C*_Eff_ of both electrodes from the half‐cell studies. In the first cycle, graphite and BP‐C show a similar initial *C*_Eff_ (≈75%), leading to the predicted electrode potentials. However, with ongoing cycling the *C*_Eff_ of the positive electrode strongly depends on the cutoff potential, as shown in Figure 4c, whereas the *C*_Eff_ of BP‐C remains also at 99% between 0.5 and 2.0 V versus Li|Li^+^ (Figure S4, Supporting Information). In the case of a cell voltage window between 2.0  and 4.7 V, the graphite positive electrode suffers from stronger parasitic reactions (lower *C*_Eff_) than the phosphorus negative electrode (*C*_Eff (graphite, 96.8%) _< *C*_Eff (BP‐C, 99%)_), which leads to Li^+^ ion trapping at the negative electrode and the described effects. For a cell with a cell voltage range of 2.0–4.3 V, the *C*_Eff_ of both electrodes is more similar (*C*_Eff (graphite, 97.8%)_ < *C*_Eff (BP‐C, 99%)_) decelerating Li^+^ ion trapping during cycling. Overall, both voltage windows lead to a stable cycling behavior over 100 cycles without any Li metal plating and no visible pulverization of particles (Figure S5, Supporting Information) demonstrating the practicability of these DIB full‐cells with reasonable capacity balancing.

#### 2.3.2 Structural Characterization of Electrodes Extracted from Cycled Cells

**Figure 7:**
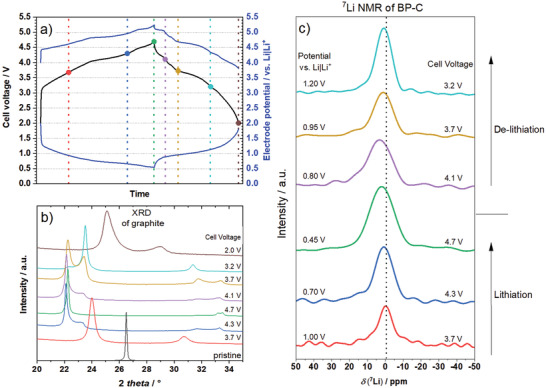
a) Cell voltage and electrode potential curves in the second cycle of a graphite || BP‐C cell (three‐electrode configuration, full‐cell setup) cycled between 2.0 and 4.7 V with 3.4 m LiTFSI in DMC as electrolyte. b) Ex situ XRD measurements of graphite cathodes and c) ex situ ^7^Li MAS NMR measurements of BP‐C anodes at a MAS rate of 25 kHz according to the corresponding SOCs marked in part a). Black dotted line in (c) corresponds to the ^7^Li chemical shift of the pristine sample soaked with electrolyte (the signal is caused by residues of the conducting salt on the electrode rather than alloying and/or intercalation).

Ex situ measurements have been employed to probe the processes in electrodes extracted from DIB full cells (**Figure** **7**). Ex situ XRD patterns of cycled graphitic cathodes were recorded at different states‐of‐charge (SOC) to investigate the structural changes during cycling. The XRD patterns are shown in Figure 7b and correspond to the cell voltages and electrode potentials marked with solid circles and dashed vertical lines in Figure 7a. The pattern of the pristine electrode is dominated by the intense graphitic (002) reflection at 26.55 °. At a cell voltage of 3.7 V, which corresponds to a positive electrode potential of ≈4.4 V versus Li|Li^+^, two new reflections at 24.01° and 30.71° arise. As reported previously for TFSI‐based GICs by Schmuelling et al., this behavior is an evidence for a staging mechanism, proposed for anion intercalation.^[^ ^50^ ^]^ The main stage index *n* as well as the gallery height *h_C‐A‐C_* can be calculated from the 2*θ* values of the most intense reflections ((00*n*+1) and (00*n*+2) (see Supporting Information for further information).^[^ ^52^ ^]^ After the formation of a stage‐3 GIC at 4.3 V, a stage‐1 TFSI‐based GIC is clearly developed with a gallery height of ≈8 Å at the highest SOC at 4.7 V (further information about the repeat distance of the unit cell (*I*_Gallery_) and the gallery height (*h_C‐A‐C_*) are shown in Table S3, Supporting Information). During discharge, the deintercalation mainly starts at the observed plateau at a cell voltage of 4.1 V and the staging mechanism progresses in reverse, ending up with the formation of a stage‐5 TFSI‐GIC at 2.0 V. The results show that the electrochemical data correlate well with the structural changes and a reversible staging mechanism expected for TFSI‐based GICs.

Ex situ ^7^Li magic‐angle spinning (MAS) nuclear magnetic resonance (NMR) spectroscopy was used to obtain additional insights into the chemical environment of the lithiated forms of the BP‐C composite. Due to the highly nanocrystalline structure of the electrode materials, only ^7^Li MAS NMR is shown here, as ^31^P MAS NMR did not deliver any specific information caused by the low mass loading of the electrodes. The chemical shift of the ^7^Li nucleus is shown at different SOCs in Figure 7c. A pristine electrode was soaked with electrolyte, dried, and measured afterward to clearly distinguish between the electrolyte‐based signal and lithiated phases. The pristine electrode showed a ^7^Li chemical shift of ‐1.7 ppm, which is caused by residues of the conducting salt on the top of the electrode (marked as dotted line in Figure 7c). During charge it can be clearly seen that the ^7^Li signal shifts to higher values, whereas it decreases again to lower values during discharge, indicating the ongoing lithiation of the active material. However, the work of Peng et al. revealed the coexistence of several Li*_x_* P alloys in the studied potential region,^[^ ^53^ ^]^ suggesting that the observed ^7^Li signal may be composed of a few co‐existing Li*_x_* P phases, which makes an unambiguous assignment of specific signals to specific Li*_x_* P phases difficult. For better insights into the mechanism of the lithiation of BP‐C composite, future experiments in the form of in situ NMR‐spectroscopy may be useful, and a calibration study of Li*_x_* P reference phases from which both the ^7^Li as the ^31^P chemical shifts can be determined may be suggested. LiP and Li_3_P phases were detectable in the ex situ XRD patterns of electrodes charged to 0.45 and 0.01 V versus Li|Li^+^ in half‐cells (Figure S6, Supporting Information). This suggests that the overall mechanism of the reaction of phosphorus in the electrodes and the observed capacity are consistent with the expected three‐electron alloying process forming a Li_3_P phase.

### 2.4 Comparison of Graphite || BP‐C and Graphite || Graphite Full‐Cells

Our results presented in the previous section demonstrate the possibility of functional DIB full‐cells that utilize the lithiation of a BP‐C composite in the negative electrode and the intercalation of anions into the graphite positive electrode. As stated in the introduction, the key promise of the implementation of negative electrodes with the (de‐)alloying reaction mechanism in DIB full‐cells is in achieving a higher specific energy in comparison to that of DGBs. In line with this goal, the experimental results of the full‐cell DIBs reported here should be compared with graphite || graphite (DGB) full‐cells. Furthermore, comparisons with other DIB full‐cells with negative electrodes incorporating materials functioning via the (de‐)alloying mechanism can be drawn as well.

**Figure 8:**
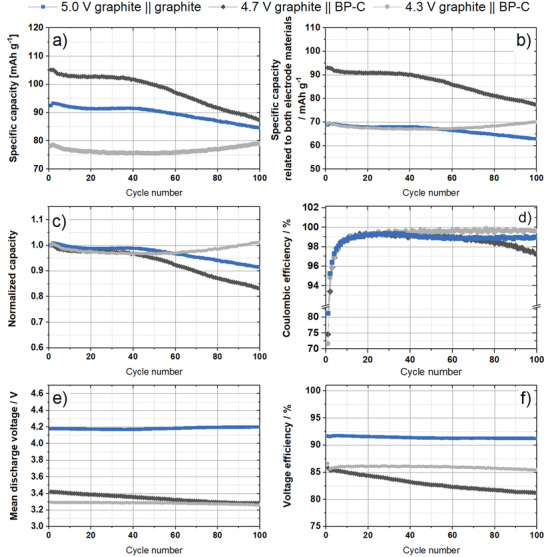
Electrochemical performance of graphite || BP‐C cells (two‐electrode configuration; full‐cell setup; cell voltage: 2.0– 4.7 V, black, and 2.0–4.3 V, gray) and graphite || graphite cells (three‐electrode configuration; full‐cell setup; 2.0–5.0 V, blue) in constant current cycling experiments: a,b) specific discharge capacities related to the mass of the positive electrode and both electrode active materials; c) measured capacity normalized to the first discharge capacity, d) Coulombic efficiency of the cells, e) mean discharge voltage, f) voltage efficiency. 3.4 m LiTFSI in DMC as an electrolyte at a current of 50 mA g^–1^ (related to the positive electrode).

To provide a fair comparison with graphite || BP‐C full‐cells (PGDIBs) and practical graphite || graphite full‐cells (DGBs), DGB cells with the same N/P ratio (1.2/1.0) were built and investigated by constant current cycling experiments. Graphite || Li metal cell studies (half‐cell setup) with graphite as negative electrode were initially performed to enable a capacity‐related balancing based on practical specific capacities (Figure S7, Supporting Information). Furthermore, two different cell voltage windows (2.0–5.0 V and 2.0–5.1 V) were evaluated for the DGB full cells. Since a higher cut‐off cell voltage of 5.1 V leads to Li metal plating after five cycles, as observed by negative electrode potentials ≤0 V versus Li|Li^+^, a cell voltage window of 2.0–5.0 V was chosen for safety reasons to prevent Li metal plating (further experimental data on the cycling of DGB cells are provided in Figures S8 and S9, Supporting Information). The constant current charge–discharge data of the two PGDIB cells (cell voltage ranges: 2–4.7 V and 2–4.3 V) and the reference DGB cell are presented in **Figure** **8**, and various performance metrics over the course of the first 100 cycles can be observed. These include specific capacity and capacity retention (Figure 8a–c), Coulombic efficiency (Figure 8d), mean discharge voltage (Figure 8e) as well as voltage efficiency (Figure 8f).

It can be seen from Figure 8a that the DGB cell achieves a lower specific discharge capacity related to the positive electrode (by ≈15%) than the BP‐C based DIB cell operated at 2.0–4.7 V. However, its capacity (related to the positive electrode only) is superior to that of the PGDIB cell operated at 2.0–4.3 V. The situation changes when the cell capacities are re‐calculated per mass of active materials on both anode and cathode (Figure 8b). The PGDIB cell (2.0–4.7 V) shows a 37% higher specific capacity than that of the DGB cell, while the PGDIB cell with the reduced voltage range (2.0–4.3 V) has a similar capacity but an improved cycling stability. The possibility to improve the specific capacity of the cell represents a major benefit of using black phosphorus in the anode and is directly related a much higher specific capacity of the BP‐C composite, which helps to reduce the total mass of active materials, lifting the value of specific capacity related to both electrodes. For further discussion of achieved capacities, see comment 1 in Supporting Information. The cycling stabilities of the three cells are best illustrated by a plot of their normalized capacities (Figure 8c), which shows that the PGDIB cell operated at 2.0–4.3 V has a better capacity retention than the DGB cell. The cause of the rising capacity after the first 50 cycles needs to be investigated further in future works. The capacity degradation was observed in both the DGB cell and PGDIB cell (2.0–4.7 V) after the first 40 cycles.

The Coulombic efficiency (*C*_Eff_) is another important characteristic of battery cells. During cycling, the *C*_Eff_ of all cells increases to 99% or above (Figure 8d), with the *C*_Eff_ of PGDIB cells operated at 2.0–4.3 V being a standout. The *C*_Eff_ of the DGB cell also remains constant although at a lower value. If a broader cell voltage range of 2–4.7 V is used for PGDIB cells, the *C*_Eff_ of the cell decreases from the 70^th^ cycle onwards to an eventual value of 97.2% in the 100^th^ cycle.

The mean discharge cell voltages for all cells are shown in Figure 8e. The graphite || BP‐C full‐cells display a lower mean discharge voltage caused by a higher delithiation potential of BP‐C with respect to that of graphite. The DGB cell shows a constant discharge voltage of ≈4.2 V, whereas the mean cell voltage of the PGDIB cell with the voltage range of 2–4.7 V changes from 3.4 V at the onset of cycling to 3.3 V after 100 cycles. The mean voltage of the PGDIB cell with the cell voltage range of 2.0– 4.3 V remains relatively stable at a level of slightly below 3.3 V. Despite their promise of achieving a higher capacity and specific energy in the cell, the inherent disadvantage of most electrode materials with the (de‐)alloying mechanism (including phosphorus) is their larger voltage hysteresis with respect to that of graphite. As expected, this leads to a lower *V*_Eff_ of the cells that use the BP‐C composite than with graphite as negative electrode material (Figure 8f). For the PGDIB cells operated at 2.0–4.7 V, the *V*_Eff_ changes from 86% in the first cycle to 81% in the 100^th^ cycle, while the *V*_Eff_ for the PGDIB cell with the voltage range of 2.0–4.3 V stays above 85%. These *V*_Eff_ values are somewhat lower than the *V*_Eff_ for the DGB cell which remains nearly constant at 92%. A further important performance indicator is the energy efficiency (*E*_Eff_), which is depicted in Figure S10d (Supporting Information) and is closely linked to the *V*_Eff_. A larger voltage hysteresis in the negative electrodes of PGDIB cells translates into somewhat lower *E*_Eff_. For example, the DGB cell shows an *E*_Eff_ of 91%, whereas the PGDIB cell with the cell voltage range of 2– 4.7 V displays a lower *E*_Eff_ of 84% in the 10^th^ cycle and 78% in the 100^th^ cycle. The *E*_Eff_ of the PGDIB cell with the cell voltage range of 2.0–4.3 V stays at the level of ≈85%, which is linked to the excellent *C*_Eff_ of this cell and its more consistent *V*_Eff_.

**Figure 9:**
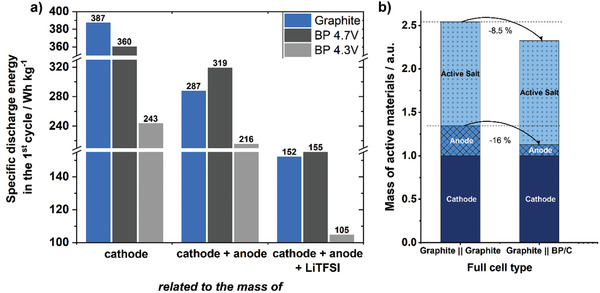
a) Specific energy of PGDIBs cells and DGB cells in the first cycle related to the mass of active materials, i.e., (i) cathode, (ii) cathode and anode, (iii) cathode, anode, and LiTFSI salt. b) Relative mass distribution of active materials of DGB and PGDIB cells. Normalized to the mass of cathode active material.

The most important metric for a battery cell is its specific energy, and the plot of specific energies in the first cycle for the three cells is presented in **Figure** **9**a. Here, the specific discharge energies of each first cycle are calculated i) per cathode active material, ii) total mass of both electrode active materials, and iii) all active materials including cathode, anode, and active LiTFSI salt. Related to the mass of cathode active material (i), the DGB cell shows a higher specific energy than all BP‐containing cells (Figure 9a). However, when the specific energy is related to the mass of cathode and anode active material (ii), the PGDIB cell with the voltage range of 2.0–4.7 V clearly outperforms the DGB in terms of specific energy. The cell shows an increased specific discharge energy of ≈319 Wh kg^–1^ compared to 287 Wh kg^–1^ for the DGB cell, which corresponds to an increase of more than 10% at the start of cycling. However, during cycling the specific energy decreases stronger for the PGDIB cell than for the DGB cell (Figure S10a–c, Supporting Information). It should be noted here that this work is the first reported attempt to fabricate a PGDIB of this nature in the literature, and we expect that the cycling stability of the cell can be improved in the future. One way to enhance the cycling stability of the PGDIB cell is to decrease its cell voltage range, and indeed a PGDIB operated at 2–4.3 V demonstrates an example of such an improvement. While the initial specific energy of this PGDIB cell (217 Wh kg^–1^) is considerably inferior to that of the DGB cell, the difference decreases after 100 cycles (values of 224 vs 264 Wh kg^–1^, respectively). In contrary to LIB cells, the conductive salt is part of the active materials in DIBs. Calculating the specific energy related to the mass of anode, cathode, and mandatory salt for the ion uptake (iii), the values as well as the benefit of BP‐C containing cells are strongly decreased (Figure 9a). This effect is caused by the high mass of TFSI^–^ anions which is demonstrated in Figure 9b. The high molecular weight of TFSI^–^ decreases the overall mass reduction by implementing high‐capacity anodes. The advantage of high‐capacity anodes can be strongly increased by switching to electrolyte formulations using lighter and smaller anions like PF_6_^–^ or BF_4_^–^ (see Figure S11, Supporting Information). The impact of the electrolyte formulation on the specific energy and energy density of DGBs has also been discussed in our previous work.^[^ ^10a^ ^]^

**Figure 10:**
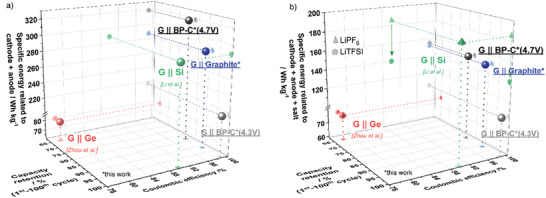
Comparison of specific discharge energy (first cycle, related to the mass of a) both electrode active materials or b) electrode active materials and active salt), capacity retention and *C*_Eff_ (10^th^ cycle) between the graphite || BP‐C cells, graphite || graphite cells and the reported DIBs in literature^[^ ^35^, ^36^ ^]^ utilizing graphite (G) as the positive electrode. For data published in literature, the specific energy, as shown in (b), was calculated with used LiPF_6_ and additionally with LiTFSI to enable a fair comparison. Further information and performance values are described in Supporting Information.

The specific discharge energies of the first cycle, *C*_Eff,_ and the experimentally demonstrated capacity retentions of the two PGDIB cells assembled in this study are compared to the values for DGBs and selected DIB full‐cells with anodes based on the (de‐)alloying mechanism reported in the literature to provide the context for the results in this study. The data are shown in **Figure** **10**. It should be noted that a comparison to other battery technologies such as LIBs should be avoided since other components (inactive materials and the electrolyte) must be considered for a fair comparison.^[^ ^10a^ ^]^ Furthermore, only lithium‐based DIBs were taken into account, since a different cation (e.g., larger cations such as Na^+^ or K^+^) even complicates the comparison and naturally leads to lower specific energies (compare Figure 9). The plotted specific energy in Figure 10a is calculated on basis of the combined masses of both electrodes. There are a few DIB studies in the literature on the cells containing a graphite‐based cathode and an anode incorporating a material functioning via the (de‐)alloying mechanism.^[^ ^35^, ^36^, ^37^, ^40^ ^]^ However, among these reports, the number of studies in which at the same time i) Li metal plating can be excluded, ii) the appropriate analysis of practical N/P‐ratios is provided, and iii) an appropriate three‐electrode setup is used to diagnose the behavior of individual electrodes, is rather small. Considering these reporting limitations, we were able to identify two studies on lithium‐based DIBs with Si‐containing electrodes^[^ ^35d^ ^]^ and Ge‐containing electrodes,^[^ ^36^ ^]^ which qualified as good comprehensive reference data points to compare with our results. The explanation on how specific energy data were calculated based on the reported cell voltages, specific capacities, and N/P‐ratios is provided in the Supporting Information. From the analysis of available cell metrics, it can be stated that our reported DIB incorporating black phosphorus (cell voltage range of 2.0–4.7 V) achieves a higher specific discharge energy related to both electrode active materials than those reported for Ge‐based,^[^ ^36^ ^]^ Si‐based^[^ ^35d^ ^]^ DIBs or DGBs analyzed in this work (Figure 10a, data point G || BP‐C (4.7 V)). When the cell voltage range of PGDIB cells is decreased to 2.0–4.3 V, its specific energy decreases; however, the cyclic stability and *C*_Eff_ appear to rival or exceed those of the DGB cell and graphite || Si cell (Figure 10a, data point G || BP‐C (4.3 V)).

As the active salt represents a reactive species in DIBs, it may be argued that the specific energy of DIBs should be recalculated to the combined mass of active materials in electrodes and active salt. The nature of the salt can also make a substantial difference in the values obtained (compare data in Figure S11, Supporting Information, for different salts). Therefore, to provide a balanced presentation, the specific energy calculations related to the mass of electrode active material and active salt are additionally presented in Figure 10b. The published cell of Li et al. outperforms the PGDIB in terms of specific energy (green triangle). However, in the study of Li et al. LiPF_6_ is used as conductive salt which benefits the specific energy compared to PGDIB including the mass of active salt. To enable a fair comparison and address the influence of the active salt, theoretical specific energy values based on LiTFSI (green circle) were calculated assuming hypothetically the same specific capacity and voltage values. Considering these assumptions and calculations, the PGDIB shows the highest reported specific energy. As it follows from the presented data, G || BP‐C PGDIBs demonstrate an encouraging electrochemical performance. A high specific energy (319 Wh kg^–1^, related to both electrode active materials, or 155 Wh kg^–1^, related to masses of electrode active materials and active salt, can be achieved, while the stability and described efficiencies of the cell can be altered by adjusting the maximum potential for the positive electrode. The capacity retention of the most energy‐dense cell is somewhat inferior at present, but we take a view that further studies and optimization of the graphite positive electrode will allow to achieve a significant improvement. A promising performance has been demonstrated for the initial prototypes of PGDIBs.

## 3 Conclusion

A composite material containing black phosphorus/carbon (BP‐C) was evaluated in this work for the first time as a high‐capacity negative electrode material for lithium‐based dual‐ion batteries (DIBs) with potential to boost the energy density and safety compared to “classical” dual‐graphite batteries (DGBs). After characterizing the electrochemical behavior of black phosphorus‐containing electrodes in highly concentrated electrolytes, novel BP‐C || graphite (PGDIB) cells with a reasonable mass and capacity balancing were designed and characterized in a three‐electrode setup. By optimizing the cell voltage, a high specific energy of 319 Wh kg^‐1,^ related to the mass of electrode active material, or 155 Wh kg^–1^, related to masses of electrode active materials and active salt, and a high Coulombic efficiency (>99%) is achieved in a cell voltage window of 2.0– 4.7 V. In turn, a PGDIB cell operated within a voltage window of 2.0–4.3 V displays superior cycling stability and Coulombic efficiency. However, regarding the specific energy including all active parts in DIBs, conductive salts with a high molecular mass reduce the benefits of high‐capacity anodes significantly, so that a suitable anode/electrolyte combination must be targeted. Mechanistic studies revealed the staging behavior in the graphite positive electrode and the formation of lithiated phosphorus alloys in the negative electrode. Thus, with this study, we introduce a novel and promising cell configuration for practical and safe high‐energy DIB cells that will stimulate the progress of DIB full‐cells toward commercial application for stationary energy storage.

## 4 Experimental Section

### Synthesis and Electrode Materials

Black phosphorus was prepared by milling six grams of commercial red phosphorus powder (Alfa Aesar, 100 mesh, 98% purity) in a stainless steel jar using a Fritsch Pulverisette 5 planetary ball mill under Ar atmosphere (200 kPa excess pressure above atmospheric pressure) for 25 h. The ball to powder weight ratio was 110:1 (ten stainless steel balls with a diameter of 25.4 mm were used) and the rotation speed of the mill was set to 200 rpm. The as obtained black phosphorus powder was milled again together with graphite (Sigma Aldrich, #282863, <20 µm) in a weight ratio of 1:1 (6 g in total) using the same milling time and conditions. All ball‐milled powders were removed from the stainless steel jar inside an Ar‐filled glove box. The powders were prepared in a procedure similar to our previous work.^[^ ^43^ ^]^ The resulting black phosphorus – carbon material is referred to as “BP‐C” in the following.

Synthetic c‐nergy KS6L flake‐type graphite (Imerys Graphite & Carbon) was used as a material for the cathodes due to its promising performance for anion intercalation in electrolytes with organic solvents, as shown in previous studies.^[^ ^15b^ ^]^ A high degree of graphitization, small particle size distribution, and high specific surface area, among other properties, lead to the optimum electrochemical performance for this commercially available graphite. SMG A4 (Hitachi) graphite was used for negative electrodes to compare the performance of BP‐C consisting full‐cells with conventional DGBs.

### Electrode and Electrolyte Preparation

All graphite‐based electrodes, both positive and negative, were composed of 90 wt% active material, 5 wt% sodium carboxymethyl cellulose (Na‐CMC) binder (Walocel CRT 2000 PPA12, Dow Wolff Cellulosics) and 5 wt% conductive carbon (C‐NERGY Super C65). For the cathode, aluminum foil (Evonik Industries, 15 µm thickness) was used as the current collector, whereas a dendritic copper foil (Schlenk) was utilized for the anode. Both graphite‐based electrodes were prepared as reported in the previous publication.^[^ ^15b^ ^]^ The mass loading of the anodes was 1.1 ± 0.1 mg cm^–2^, and that of the cathodes was 3.0 ± 0.1 mg cm^– 2^. Further explanations to the mass ratio and capacity balancing of both electrodes are given in the results part.

Black phosphorus–carbon composite electrodes were prepared using 80 wt% synthesized BP‐C composite (weight ratio 1:1), 10 wt% sodium carboxymethyl cellulose (Na‐CMC) binder (Walocel CRT 2000 PPA12, Dow Wolff Cellulosics) and 10 wt% conductive carbon (C‐NERGY Super C65). First, the ball‐milled BP‐C‐composite was dry mixed with Na‐CMC binder in an Ar‐filled glove box. Subsequently, the dry mixture was transferred out of the glove box and distilled water was added quickly. After that, the suspension was stirred in a small jar until the binder was dissolved. In the final step of paste fabrication, the conductive agent was added and the mixture was stirred overnight. The electrode paste was cast on dendritic copper foil using an automatic film applicator at a speed of 50 mm s^–1^. The electrode sheets were dried at 80 °C under atmospheric pressure overnight. In the next step, disks with a diameter of 12 mm were cut out and dried again under reduced pressure (10^–2 ^mbar) at 80 °C overnight. The average mass loading of the BP‐C electrodes was 0.35 mg ± 0.05 mg cm^– 2^ depending on the balancing and mass ratio in the investigated DIB full cells. The highly concentrated electrolytes, 4 m LiPF_6_ (BASF) and 3.4 m LiTFSI (BASF) in dimethyl carbonate (DMC, BASF), were prepared as reported in previous studies.^[^ ^25^, ^32^ ^]^ Conducting salts were dried at a reduced pressure (10^–3 ^mbar) at 80 °C.

### Assembly of Electrochemical Cells

In the manuscript, all different types of cells are described by the following nomenclature, according to Nölle et al.:^[^ ^54^ ^]^ Positive electrode || Negative electrode.

Electrochemical measurements were performed in either stainless steel two‐electrode coin cells (2032‐type) or stainless steel three‐electrode T‐type cells (Swagelok). For the two‐electrode coin cells Li metal and BP‐C containing electrodes were used as negative and positive electrode (ø = 12 mm) respectively to investigate different electrolytes. The electrodes were separated by a glass microfiber separator (Whatman filter, grade GF/D, ø = 13 mm), which was soaked with 120 µL of the corresponding electrolyte. Three‐electrode T‐type cells consisting of a Li metal reference electrode (RE; ø = 5 mm) and anode/cathode (ø = 12 mm; full‐cell setup; control of cell voltage) or counter (CE)/working (WE) electrodes (ø = 12 mm; half‐cell setup; control of WE potential) were used to additionally monitor the electrode potentials during cycling and to investigate the C‐rate performance.^[^ ^54^ ^]^ In a half‐cell configuration, Li metal was used as the CE and the WE potential was controlled via the RE. Glass microfiber separators (Whatman filters, grade GF/D, ø = 13 mm between WE and CE; ø = 8 mm for the RE) were soaked with 120 µL and 80 µL of the corresponding electrolyte, respectively. A Mylar foil (DuPont) was used to prevent contact between the current collectors and the cell body.

For the long‐term cycling and comparison of the key performance indicators (KPIs) of graphite || BP‐C and graphite || graphite (DGB) full‐cells stainless steel two‐electrode coin cells (2032‐type) were used. Therefore, 12 mm electrodes were used for the anodes and cathodes separated by a glass microfiber filter (Whatman; grade GF/D, ø = 13 mm) which was soaked with 120 µL of the corresponding electrolyte.

### Electrochemical Charge—Discharge Experiments

Constant current charge–discharge cycling was performed on a Maccor 4000 battery test system at 20 °C. For investigations of the electrolyte on the performance of BP‐C || Li metal cells (full‐cell setup), electrodes were cycled at 0.1 C for five formation cycles followed by 100 cycles at 0.2 C. The theoretical capacity (1C = 1484 mAh g^–1^) of BP‐C electrodes was calculated on the assumption of the formation of Li_3_P and LiC_6_ phases. The specific currents and capacities were set and calculated per total mass of BP‐C composite. For rate capability studies, the BP‐C || Li metal half‐cells were cycled with a specific current of 500 mA g^–1^ for 20 cycles followed by currents of 100, 200, 500, 1000, 2000, and 500 mA g^–1^ used for five cycles each. This study was controlled by the WE potential (BP‐C electrode) with cut‐off limits of 0.01 V and 2 V versus Li|Li^+^.

The SMG A4 graphite WE was initially cycled with 100 mA g^–1^ for 20 cycles in SMGA4 graphite || Li metal cells; this was followed by currents of 20, 50, 100, 200, 400, and eventually 100 mA g^–1^ for five cycles each. The WE potential was controlled between 0.01 and 1.5 V versus Li|Li^+^. The KS6L graphite WE was cycled at 50 mA g^–1^ for 20 cycles in KS6L graphite || Li metal cells followed by currents of 10, 20, 50, 100, 200 and eventually 50 mA g^–1^ for five cycles. The WE potential was controlled between 3.4 V versus Li|Li^+^ and different upper cutoff potentials (4.8; 5.0 or 5.2 V vs Li|Li^+^).

Long‐term charge–discharge cycling experiments were conducted at 50 mA g^–1^ with regard to the cathode without any formation cycles and with cell voltages limited between 2 V and an appropriate cutoff voltage (4.3 V or 4.7 V) to evaluate the full‐cell performance. In the absence of a separate explanation, the capacity in all graphs of full‐cell data is related to the mass of the graphite cathode. For energy calculations, only the sum of the active masses of both electrodes was considered. Specific energy calculations were performed by the MACCOR Battery Tester integrating voltage course over specific capacity. Voltage efficiency values were calculated by dividing the mean discharge voltage by mean charge voltage.^[^ ^41^, ^55^ ^]^

### Material Characterization

X‐Ray diffraction (XRD) patterns of the phosphorus–carbon composite material were obtained using a PANalytical Empyrean instrument fitted with a Cu K*α* radiation (*λ* = 1.54181 Å) source. The pattern was collected using a step size and a step time of 0.02° and 398 s, respectively. The as‐obtained pattern was analyzed using X'Pert High Score Plus software.

Transmission electron microscopy (TEM) characterization was conducted using a JEOL JEM 2100F instrument with 200 kV accelerating voltage and equipped with an energy‐dispersive X‐ray (EDX) spectrometer; the samples were deposited onto copper grids covered with a holey formvar film from ultrasonicated ethanol suspensions. The elemental maps were acquired in the scanning TEM mode.

In order to conduct quantitative elemental analysis of the BP‐C material by EDX spectroscopy in a scanning electron microscope, the powders were pressed into a pellet using a vertical pellet press with a tungsten carbide die. The resulting pellet was then attached to an SEM stub using double‐sided carbon tape. The elemental analyses were performed at 15 kV at a probe current of 0.6 nA, using a Hitachi 4300 FESEM equipped with an Oxford energy dispersive spectrometer and Oxford INCA quantitative software. Well‐characterized reference standards were used for all relevant elements to calibrate the system prior to analysis. PAP matrix corrections^[^ ^56^ ^]^ were applied throughout to account for the differences between the reference standards and unknowns.

KS6L graphite cathodes and BP‐C anodes were examined via ex situ XRD and solid‐state ^7^Li magic‐angle spinning (MAS) nuclear magnetic resonance (NMR) techniques to investigate the storage mechanism inside the full‐cell. For mechanism assessment, the cells were cycled for one charge–discharge cycle and stopped in the second cycle at a certain voltage. Afterward, the cells were disassembled in an argon‐filled glove box and the electrodes were transferred into a vacuum‐sealed XRD sample holder with a dome to avoid any contact with moisture. The crystal structures in the intercalated graphite and partially lithiated BP‐C electrodes were evaluated in this case on a Bruker D8 Advance diffractometer with Cu K_*α*_ radiation (1.54 Å). The diffraction patterns of graphite were recorded in the *2θ* range of 10–60° at a scan rate of 0.021° per step and a step time of 2 s. The diffraction patterns of BP‐C were recorded in the *2θ* range of 10–55° at a scan rate of 0.021° per step and a step time of 4 s. The sample preparation for the solid‐state ^7^Li MAS NMR measurements, including grinding, diluting, and packing the samples into zirconium dioxide (ZrO_2_) rotors, was performed in an argon‐filled glove box. The electrode paste was scratched from the Cu current collector using a ceramic scalpel, ground, and diluted 1:4 by weight with MgO to prevent eddy currents and thereby additional heating of the sample during the NMR measurement. All ^7^Li MAS NMR spectra were recorded on a 200 MHz Bruker DSX spectrometer equipped with a 4.70 T wide bore magnet at *ν*_L_(^7^Li) = 77.8 MHz using a 2.5 mm MAS probe (Bruker VTN design). The ^7^Li chemical shifts were referenced to a 1 m LiCl solution and its isotropic chemical shift was set *δ*_iso_  =  0 ppm. For the investigation of the Li species, single‐pulse ^7^Li MAS NMR experiments were performed with a recycle delay of 60 s to ensure full T_1_ relaxation after each transient. A flip angle of *π*/2 for a nutation frequency of 125 kHz was used for the ^7^Li measurements.

The investigation of the surface morphology of the pristine and cycled graphite and BP‐C electrodes were performed by a Zeiss Auriga scanning electron microscope (SEM, accelerating voltage: 3 kV, working distance: 3.8 mm). The corresponding cells were disassembled in a glove box after 100 cycles and the electrode surface were rinsed with 1 ml DMC. After that, the washed electrodes were dried under reduced pressure for a short period and transferred into the SEM device.

## Conflict of Interest

The authors declare no conflict of interest.

## Supporting information

Supporting Information

## Data Availability

The data that support the findings of this study are available from the corresponding author upon reasonable request.
