# Supplementary material for: Advanced Dual‐Ion Batteries with High‐Capacity Negative Electrodes Incorporating Black Phosphorus
Source: Adv Sci (Weinh). 2022 Apr 27;9(20):2201116. doi: 10.1002/advs.202201116 (PMC9284155; doi:10.1002/advs.202201116)
Supplement: Supplementary file 1 — Supporting Information [file ADVS-9-2201116-s001.pdf]

ADVANCED SCIENCE
Open Access

Supporting Information

for *Adv. Sci.*, DOI 10.1002/advs.202201116

Advanced Dual-Ion Batteries with High-Capacity Negative Electrodes Incorporating Black Phosphorus

Jens Matthies Wrogemann, Lukas Haneke, Thrinathreddy Ramireddy, Joop Enno Frerichs, Irin Sultana, Ying Ian Chen, Frank Brink, Michael Ryan Hansen, Martin Winter, Alexey M. Glushenkov and Tobias Placke**

Supporting Information

Advanced Dual-Ion Batteries with High-Capacity Negative Electrodes Incorporating Black Phosphorus

Jens Matthies Wrogemann [1], Lukas Haneke [1], Thrinathreddy Ramireddy [2], Joop Enno Frerichs [3], Irin Sultana [4,5], Ying Chen [4], Frank Brink [6], Michael Ryan Hansen [3], Martin Winter [1,7], Alexey M. Glushenkov [2,], Tobias Placke [1,*]*

[1] University of Münster, MEET Battery Research Center, Corrensstraße 46, 48149 Münster, Germany

[2] Research School of Chemistry, The Australian National University, ACT 2601, Canberra, Australia

[3] University of Münster, Institute of Physical Chemistry, Corrensstraße 28/30, 48149 Münster, Germany

[4] Institute for Frontier Materials, Deakin University, VIC 3216, Waurn Ponds, Australia

[5] School of Materials and Energy, Guangdong University of Technology, Guangzhou 51006, Guandong, P.R. China

[6] Centre for Advanced Microscopy, The Australian National University, ACT 2601, Canberra, Australia

[7] Helmholtz Institute Münster, IEK-12, Forschungszentrum Jülich GmbH, Corrensstraße 46, 48149 Münster, Germany

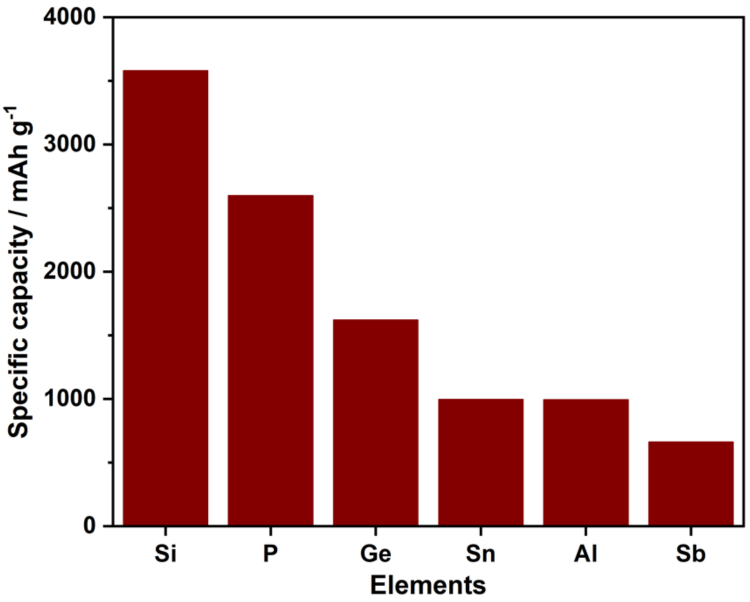

Figure S1. Theoretical specific capacities of selected materials capable of electrochemical alloying with Li^+ ions.

Additional results

Table S1. Elemental analysis of synthesized BP-C composite via energy-dispersive X-ray spectroscopy. Weight ratios of observed elements.

**Element**	**Weight ratio / %**	
	Average	St. Dev
C	47.84	1.14
O	5.05	0.29
Na	0.17	0.05
Mg	0.10	0.03
Si	0.08	0.05
P	44.94	0.42
Cl	0.22	0.05
Ar	0.17	0.05
Cr	0.41	0.11
Fe	1.79	0.45
W	1.12	0.13
Total	101.89	

Table S2. Specific de-lithiation capacities and Coulombic effiencies of BP-C || Li metal cells (two-electrode configuration; voltage range: 0.01V – 2V) for different electrolyte solutions.

**Electrolyte**	**De-lithiation capacity** 1st cycle (mAh g^{-1})	6th cycle (mAh g^{-1})	105th cycle (mAh g^{-1})	**Capacity retention (6th – 105th cycle, %)**	**Coulombic efficiency (%)** 1st cycle	6th cycle	105th cycle
**1M $LiPF_6$ in EC:EMC, 3:7 by wt.**	1194	1070	792	74.0	79.7	98.0	99.7
**3.4M LiTFSI in DMC**	1060 ± 39	897 ± 40	745 ± 32	83.0	72.9 ± 0.03	98.3 ± 0.06	99.6± 0.04
**4M $LiPF_6$ in DMC**	1009 ± 98	957 ± 134	706 ± 99	73.0	43.5 ± 1.5	94.6 ± 0.5	96.7 ± 0.4

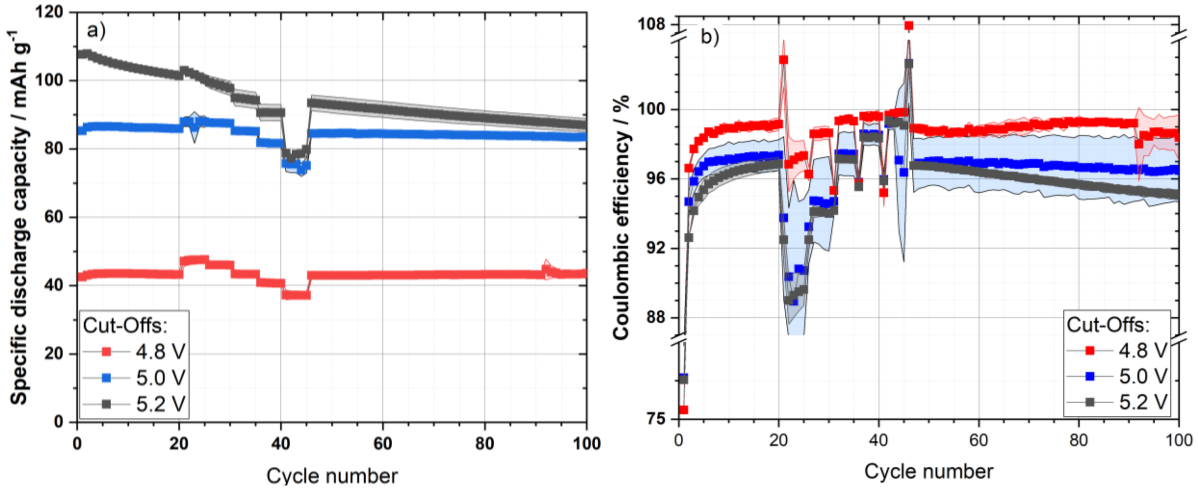

Figure S2. Electrochemical performance of graphite as positive electrode material in constant current cycling charge/discharge experiments in graphite || Li metal cells (three-electrode configuration; half-cell setup) over 100 cycles: a) Specific discharge capacity and b) C_{Eff} of a graphite WE at various specific currents and varying upper-cut-off potentials (vs. Li|Li^+) with 3.4M LiTFSI in DMC as electrolyte in a three electrode setup (same as shown in Figure 5b,c).

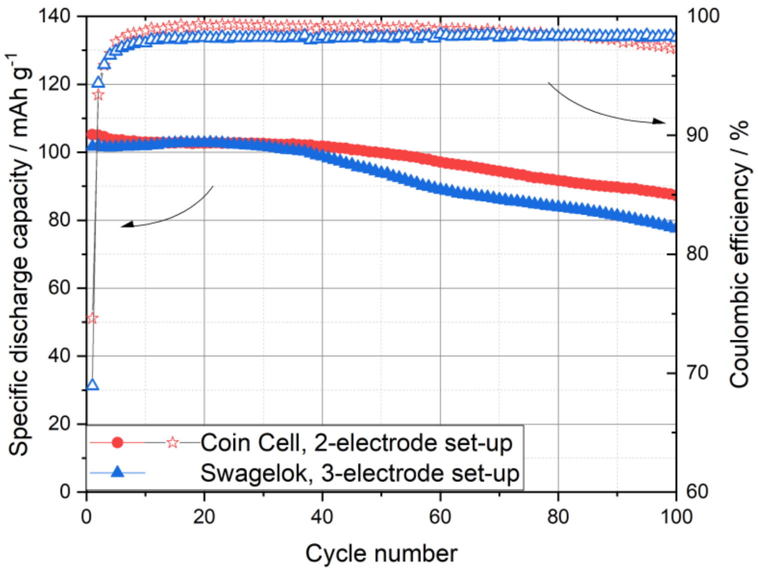

Figure S3. Comparison of the electrochemical performance of graphite || BP-C cells (two-electrode and three-electrode configuration; both in full-cell setup) in constant current cycling charge/discharge experiments with 3.4M LiTFSI in DMC as electrolyte. Cell voltage range: 2.0 - 4.7 V. Specific capacity is related to the mass of the positive electrode.

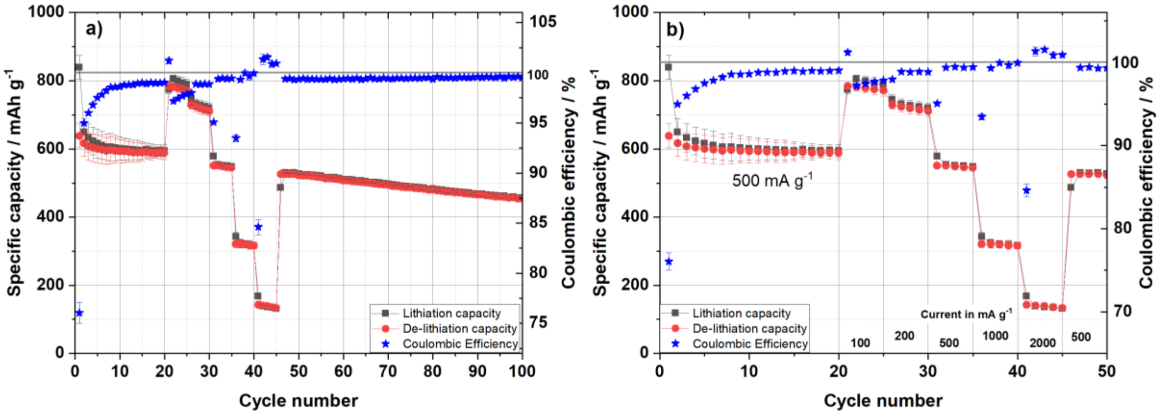

Figure S4. Electrochemical performance of BP-C composite in constant current cycling (de-)lithiation experiments in BP-C || Li cells (three-electrode configuration; half-cell setup) at various specific currents with 3.4M LiTFSI in DMC as electrolyte. Potential range: 0.5 V – 2.0 V *vs*. Li|Li$^+$. (b) magnification of (a).

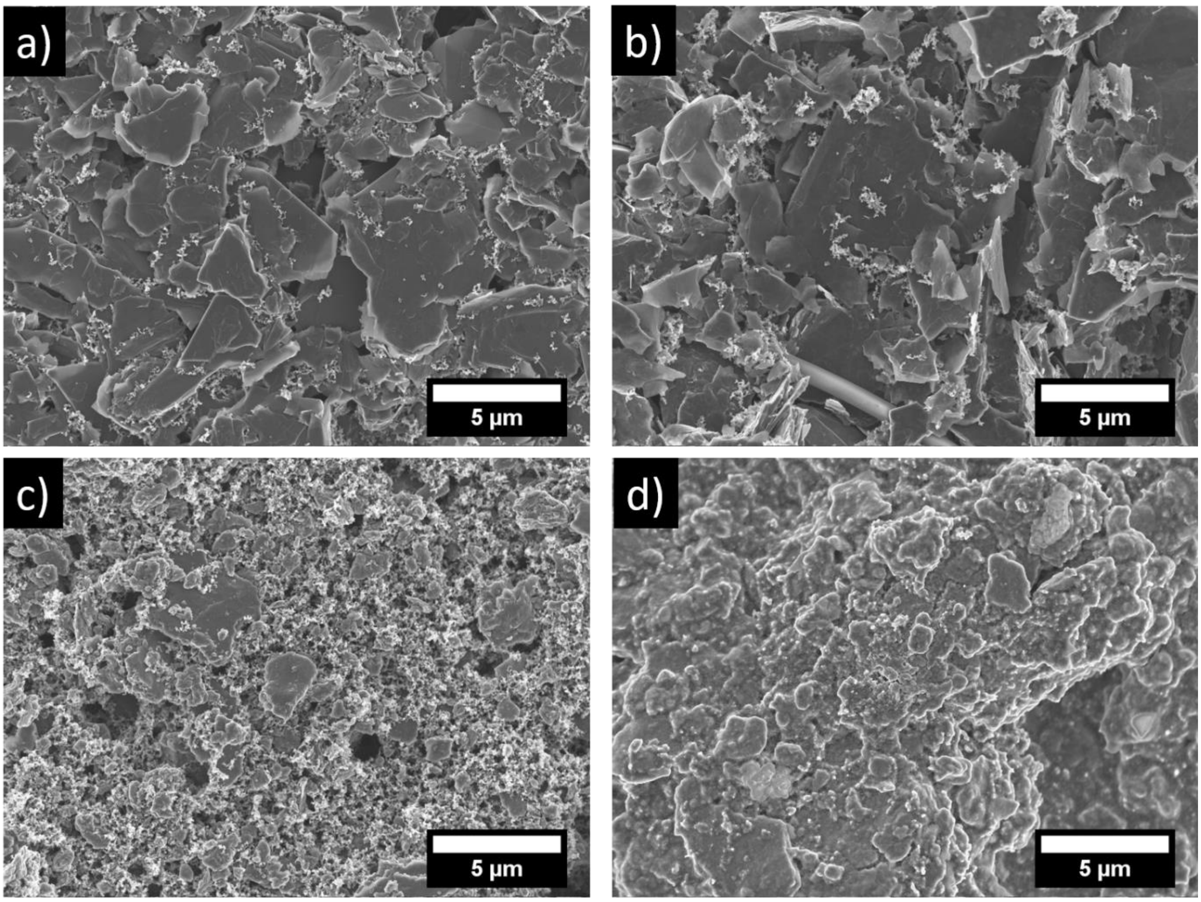

Figure S5. SEM images of a pristine graphite electrode (a), pristine BP-C electrode (c) and cycled graphite electrode (b) as well as BP-C (d) electrode after 100 cycles cycled between 2-4.7V in graphite || BP-C full-cells.

Ex situ XRD of graphite positive electrodes

During anion intercalation of anions into layered structures the arise of (00l)-reflections (with l = 1, 2, …etc.) demonstrates the staging behavior, which is caused by the repeat unit in c-direction, whereas for anion with the size of $TFSI^-$ or PF_6^- the (00n+1) and (00n+2)-reflections are the most dominant ones.[1] Since the repeat distance of the unit cell $I_{Gallery}$ is the same for both reflections, the following equation can be written:

$$I_{Gallery} = (n+1)d_{00n+1} = (n+2)d_{00n+1} \qquad (1)$$

where d_{00n} is the calculated interlayer distance, which can be calculated by Bragg´s law:

$$d_{00n+1} = \frac{\lambda}{2\,\sin\theta_{00n+1}} \qquad (2)$$

where λ describes the X-ray length and θ the Bragg angle. By substitution of (2) in (1) the dominating stage index *n* can be calculated by:

$$n = \frac{1}{1-\frac{\sin\theta_{00n+1}}{\sin\theta_{00n+2}}} - 2 \qquad (3)$$

As one unit cell of a GIC consists out of (*n*-1) stacked graphene planes, $I_{Gallery}$ can be described by

$$I_{Gallery} = h_{C-A-C} + (n-1)\ 3.35\ \text{Å} \qquad (4)$$

where h_{C-A-C} describes the gallery height of two intercalated graphene planes. Based on that following values in **Table S3** can be calculated.

Table S3. List of calculated parameters from the *ex situ* XRD measurement of graphite electrodes extracted from graphite || BP-C cells (three-electrode configuration, full-cell setup; **Figure 9b**).

**Mode**	**Cell voltage**	**$2\theta_{00(n+1)}$ / °**	**$2\theta_{00(n+2)}$ / °**	**Calc. Stage**	**$I_{Gallery}$ / Å**	**h_{C-A-C} / Å**
Charge	**3.7 V**	24.00	30.71	2.66	13.54	7.99
Charge	**4.3 V**	22.09	33.28	1.02	8.12	8.05
Charge	**4.7 V**	22.25	33.49	1.03	8.095	7.99
Discharge	**4.1 V**	22.14	33.26	1.04	8.18	8.07
Discharge	**3.7 V**	22.23	33.39	1.042	8.15	8.01
Discharge	**3.2 V**	23.51	31.35	2.07	11.59	8.02
Discharge	**2.0 V**	25.10	28.97	5.62	23.44	7.98

Ex situ ^{7}Li *MAS NMR*

Table S4. List of ^{7}Li chemical shifts measured *via ex situ* ^{7}Li NMR measurement of BP-C electrodes (**Figure 9c**).

Cell voltage	Mode	Potential vs. Li|Li^{+} / V	δ_{iso} / ppm
**Pristine**	-	-	-1.7
**3.7 V**	Charge	1.00	-1.0
**4.3 V**	Charge	0.70	1.0
**4.7 V**	Charge	0.45	2.4
**4.1 V**	Discharge	0.8	2.8
**3.7 V**	Discharge	0.95	1.3
**3.2 V**	Discharge	1.2	1.0

Ex situ XRD of BP-C electrodes

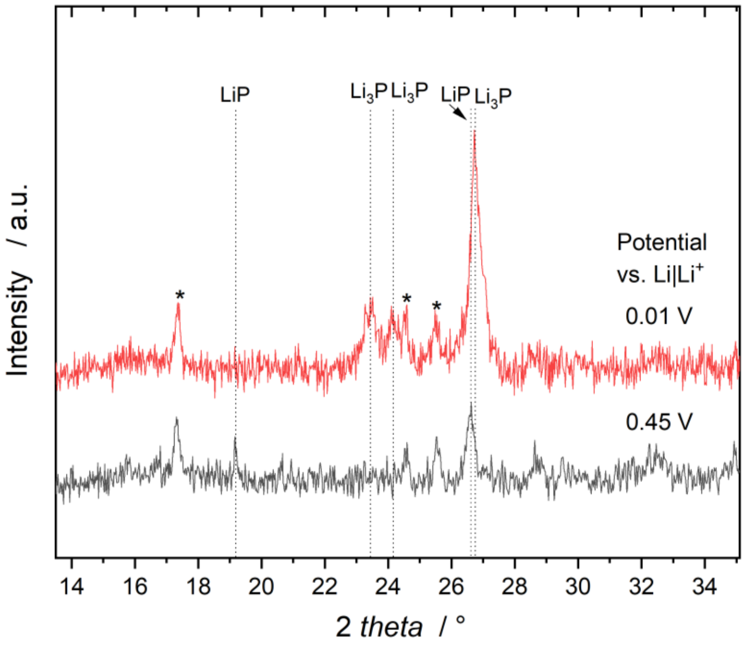

Figure S6. *Ex-situ* XRD pattern of the lithiated black P-carbon composite electrode at different potentials: 0.45 (corresponding cell voltage in full cell: 4.7 V, black) and 0.01 V *vs.* Li|Li^{+}(fully lithiated, red). The reflections of crystalline phases of LiP and Li_3P are clearly visible. The reflections marked with asterisk could not be assigned to phases expected in the electrodes and may originate from side reactions or from non-reactive components of the *ex situ* setup.

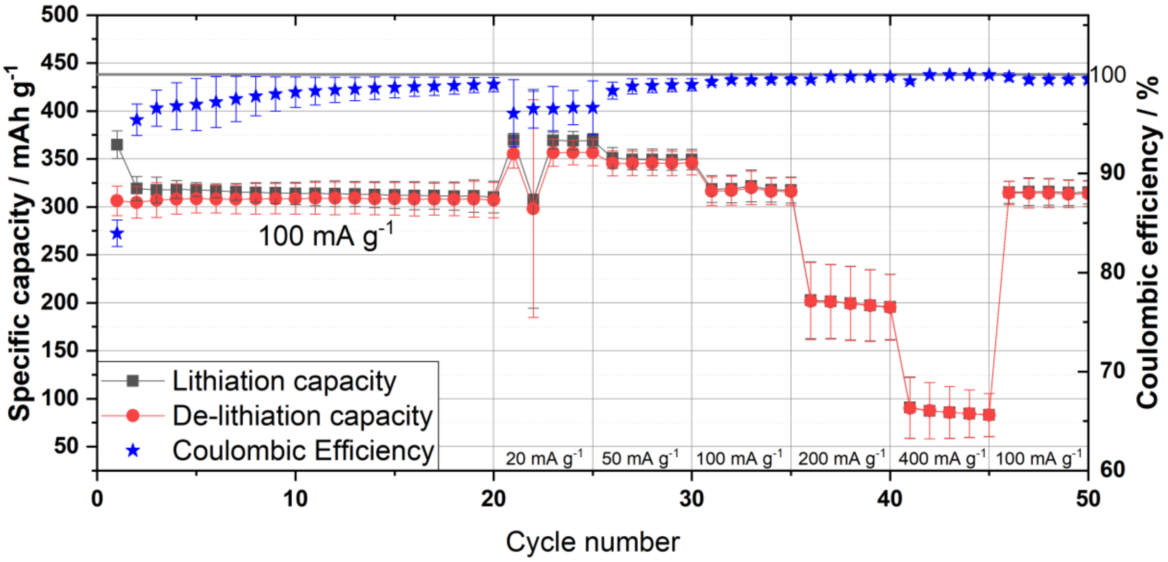

Figure S7. Electrochemical performance of graphite negative electrodes in constant current cycling lithiation/de-lithiation experiments in graphite || Li metal cells (three-electrode configuration; half-cell setup) at various specific currents with 3.4M LiTFSI in DMC as electrolyte in a potential range of 0.02 – 1.5 V *vs.* Li|Li$^+$.

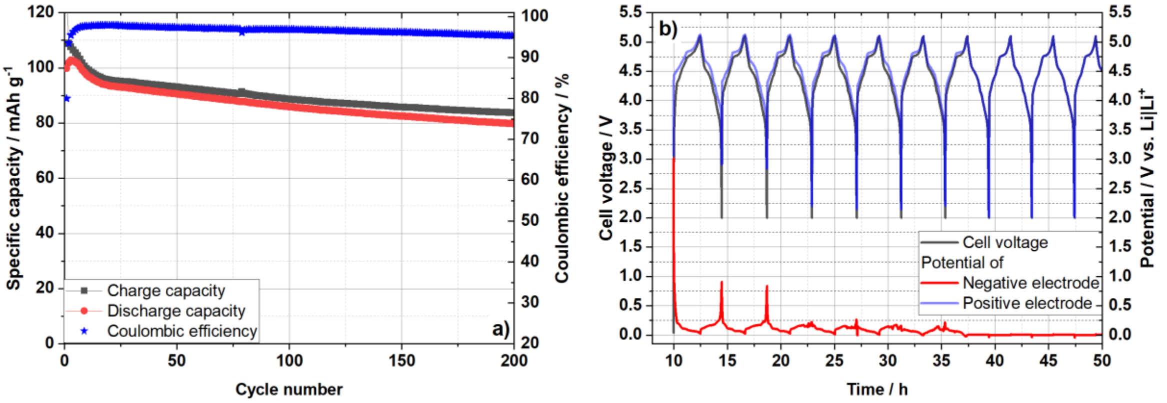

Figure S8. Electrochemical performance of graphite || graphite cells (three-electrode configuration; full-cell setup) in constant current cycling charge/discharge experiments with 3.4M LiTFSI in DMC as electrolyte in a cell voltage range of 2.0 – 5.1 V. (a) Specific charge and discharge capacity related to the mass of the positive electrode and $C_{Eff;}$ (b) respective cell voltage and electrode potentials in the first 10 cycles of the cell shown in (a).

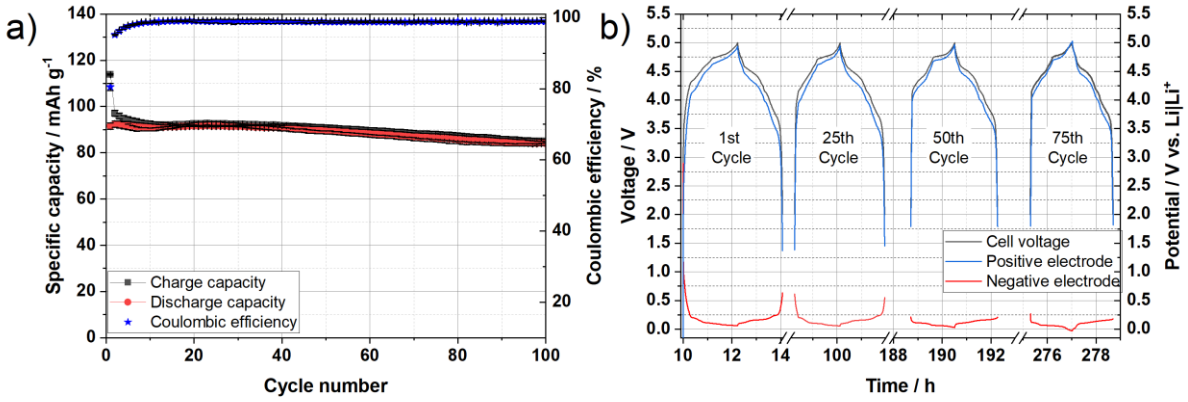

Figure S9. Electrochemical performance of graphite || graphite cells (three-electrode configuration; full-cell setup) in constant current cycling charge/discharge experiments with 3.4M LiTFSI in DMC as electrolyte in a cell voltage range of 2.0 – 5.0 V. (a) Specific charge and discharge capacity related to the mass of the positive electrode and $C_{Eff;}$ (two-electrode setup) (b) cell voltage and electrode potentials in different cycles.

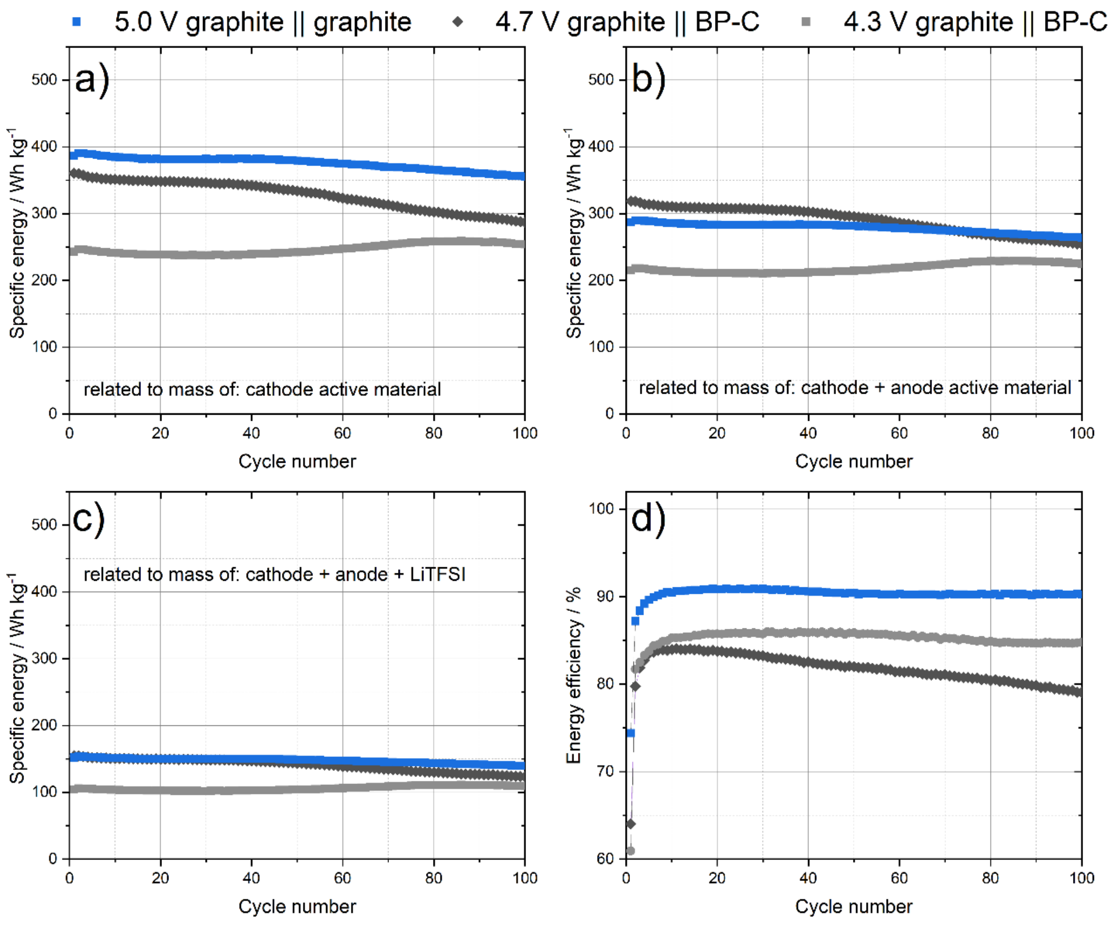

Figure S10. Specific energy of graphite || BP-C cells (two-electrode configuration; full-cell setup; cell voltage: 2.0 – 4.7 V, black, and 2.0 – 4.3 V, grey) and graphite || graphite cells (three-electrode configuration; full-cell setup; 2.0 – 5.0 V, blue) in constant current cycling experiments: Specific discharge energy related to the mass of a) the positive electrode active material; b) both electrode active materials; c) both electrode active material and mandatory LiTFSI salt for ion uptake. d) Energy efficiency. 3.4M LiTFSI in DMC as an electrolyte at a current of 50 mA g^{-1} (related to the positive electrode).

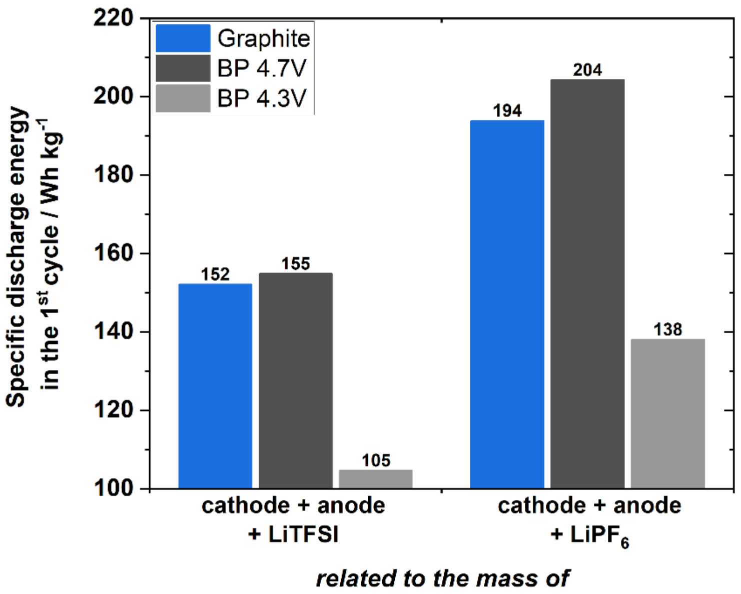

Figure S11. Specific energy of PGDIBs cells and DGB cells in the first cycle related to the mass of cathode, anode and different active lithium salts. Practical values of LiTFSI cells were calculated with $LiPF_6$ molar mass to show the impact of the used lithium salt on the specific energy.

In **Figure 10** the specific energy, C_{Eff} and capacity retention of different reported GDIBs are compared. **Table S5** shows the values which were taken from the respective study to calculate the specific energy per total electrode active mass.

Table S5. Overview of considered values for **Figure 10** based on reported studies.

**Cell chemistry**	**Reported capacity $C_{reported}$**	**Factor R [including active salt]**	**Mean discharge voltage $U_{mean\ discharge}$**	**Calculated specific energy (including active salt)**	**C_{Eff} (10th cycle)**	**Capacity 100th cycle**	**Capacity retention**
**Graphite || Ge**[2a]	281 mAh g^{-1} (per mass of N)	0.07692 (1/13) [$LiPF_6$: 0.0733; LiTFSI:0.07044]	3.7 V	79.97 Wh kg^{-1} (73.24 Wh kg^{-1})	78%	182 mAh g^{-1}	64.8 %
**Graphite || Si**[2b] **(5.0V)**	78.2 mAh g^{-1} (per mass of P)	0.891 („CB 2.2“) [$LiPF_6$: 0.569; LiTFSI:0.431]	4.15 V	289 Wh kg^{-1} (139.87 Wh kg^{-1})	90 %	78 mAh g^{-1}	99.7 %

The specific energy per total mass of active material was calculated by:

$$E = C_{reported} * R * U_{mean\ discharge} \quad (6)$$

where R describes the factor to calculate the specific capacity per total active mass, which was calculated based on the given mass ratio (N/P for Ge: 1/12 and for Si:2.2/1).[2]

For including the active salt in calculations, a XC_{20} – GIC (X: PF_6^- or $TFSI^-$) was used. In case of Graphite || Si,[2b] the following formula and values for R were used to calculate the specific capacity including anode, cathode and active salt:

$$R = \left(\frac{1}{1 + \frac{1}{\left[\frac{\left(\frac{spec.\ Capacity\ (Anode)}{Balancing} \right)}{spec.\ Capacity\ (Cathode)} \right]} + \left(\frac{\left(\frac{1}{M(C)} \right)}{Number\ C\text{–}Atoms\ for\ GIC} * n(Number\ of\ Anions\ per\ GIC) * M\ (Salt) \right)} \right) \quad (7)$$

$$R\ (Si,\ LiTFSI) = \left(\frac{1}{1 + \frac{1}{\left[\frac{\left(\frac{2248}{2.2} \right)}{125} \right]} + \left(\frac{\left(\frac{1}{12} \right)}{20} * 287.09 \right)} \right)$$

Comment 1:

As discussed, a somewhat smaller capacity (per mass of cathode) has been achieved in DGB cells compared to the capacity in the PGDIB cell operated at 2.0 – 4.7 V. This achieved specific capacity per mass of the cathode plays an important role in defining several important metrics including the specific energy, C_{Eff} and cycling stability. Upon a closer inspection, it is visible that a lower maximum cathode potential (≈5.0 V *vs*. Li|Li^+) is reached in the DGB cell than the maximum potential in the case of the graphite || BP-C cell, leading naturally to a lower capacity (see **Figure S9**). However, sticking to the chosen N/P ratio, a higher cathode potential was practically not achievable since an increased cut-off cell voltage (5.1 V) for the DBG cell leads to Li metal plating on the anode (see **Figure S8**). This effect of Li metal plating can be explained by a stronger difference in the C_{Eff} of anode and cathode in the first cycle for the two types of cells (DGB and PGDIB). In PGDIB cells the anode and cathode show nearly the same initial C_{Eff} (≈75%, see **Figure 2** and **Figure 4**), while the anode and cathode in DGB cells show a difference of ≈10% in the intial C_{Eff} (graphite anode: 85%; graphite cathode: 75%; refer to a comparison of **Figure S7** and **Figure 4**). This leads to accelerated Li^+ ion trapping in the anode and therefore, to faster Li metal plating. To counteract this drawback of the DGB cell and inhibiting Li metal plating, a different N/P ratio in accordance with the cell voltage and other requirements can be adapted. If the N/P ratio is therefore increased, this will affect multiple cell metrics as the total mass of active materials is changed and in turn, increase the benefit of high-capacity anodes with lowered mass.

References

[1] a) J. Dahn, *Physical Review B* **1991**, *44* (17), 9170; b) X. Zhang, N. Sukpirom, M. M. Lerner, *Materials research bulletin* **1999**, *34* (3), 363; c) G. Schmuelling, T. Placke, R. Kloepsch, O. Fromm, H.-W. Meyer, S. Passerini, M. Winter, *Journal of power sources* **2013**, *239*, 563; d) D. Billaud, A. Pron, F. L. Vogel, *Synthetic Metals* **1980**, *2* (3-4), 177; e) L. Haneke, J. E. Frerichs, A. Heckmann, M. M. Lerner, T. Akbay, T. Ishihara, M. R. Hansen, M. Winter, T. Placke, *Journal of the Electrochemical Society* **2020**, *167* (14), 140526.

[2] a) J. Zhou, Y. Zhou, X. Zhang, L. Cheng, M. Qian, W. Wei, H. Wang, *Nanoscale* **2020**, *12* (1), 79; b) C. Li, Y. Ju, H. Yoshitake, M. Yoshio, H. Wang, *Materials today energy* **2018**, *8*, 174.
